# Transcriptomics and co-expression networks reveal tissue-specific responses and regulatory hubs under mild and severe drought in papaya (*Carica papaya* L.)

**DOI:** 10.1038/s41598-018-32904-2

**Published:** 2018-09-28

**Authors:** Samuel David Gamboa-Tuz, Alejandro Pereira-Santana, Jesús Alejandro Zamora-Briseño, Enrique Castano, Francisco Espadas-Gil, Jorge Tonatiuh Ayala-Sumuano, Miguel Ángel Keb-Llanes, Felipe Sanchez-Teyer, Luis Carlos Rodríguez-Zapata

**Affiliations:** 1 Biotechnology Unit, Yucatan Center for Scientific Research (CICY), 97205 Merida, Yucatan, Mexico; 2 Plant Biochemistry and Molecular Biology Unit, Yucatan Center for Scientific Research (CICY), 97205 Merida, Yucatan, Mexico; 3 IDIX S.A. de C.V., Av. Sonterra 3035 int. 26, Querétaro, Mexico; 4 Polytechnic University of Huatusco, 94100 Veracruz, Mexico

## Abstract

Plants respond to drought stress through the ABA dependent and independent pathways, which in turn modulate transcriptional regulatory hubs. Here, we employed Illumina RNA-Seq to analyze a total of 18 cDNA libraries from leaves, sap, and roots of papaya plants under drought stress. Reference and *de novo* transcriptomic analyses identified 8,549 and 6,089 drought-responsive genes and unigenes, respectively. Core sets of 6 and 34 genes were simultaneously up- or down-regulated, respectively, in all stressed samples. Moreover, GO enrichment analysis revealed that under moderate drought stress, processes related to cell cycle and DNA repair were up-regulated in leaves and sap; while responses to abiotic stress, hormone signaling, sucrose metabolism, and suberin biosynthesis were up-regulated in roots. Under severe drought stress, biological processes related to abiotic stress, hormone signaling, and oxidation-reduction were up-regulated in all tissues. Moreover, similar biological processes were commonly down-regulated in all stressed samples. Furthermore, co-expression network analysis revealed three and eight transcriptionally regulated modules in leaves and roots, respectively. Seventeen stress-related TFs were identified, potentially serving as main regulatory hubs in leaves and roots. Our findings provide insight into the molecular responses of papaya plant to drought, which could contribute to the improvement of this important tropical crop.

## Introduction

Drought threatens the productivity and survival of agricultural crops worldwide. To cope with drought stress different plant tissues, such as leaves, roots, and sap, implement general and specific responses. Leaves are the main photosynthetic organs, and regulate water loss through transpiration. Upon drought stress photosynthesis decays^1^, stomata are closed^2^, and cuticle waxes are deposited^3,4^ in order to reduce excessive water loss. Roots regulate water and nutrient uptake, they are the first organs to detect water deficit, and additionally they transmit signals to the aerial parts^5,6^. Under drought stress, roots modify their architecture to increase water uptake^7^, and they increase the biosynthesis of suberin to regulate water traffic between plant and soil^8^. Upon drought stress many protein-coding genes, some of which are involved in abiotic stress tolerance, are regulated by the Abscisic Acid (ABA)-dependent and the ABA-independent signaling pathways^9^. These genes can be classified into functional and regulatory genes^10^. Functional genes include those that perform specific cellular functions such as: late embryogenesis abundant (LEA) proteins, heat shock proteins (HSP), reactive oxygen species (ROS), scavenging enzymes, osmoprotectant synthetizing enzymes, among many others. While regulatory genes control the expression and/or activity of other genes, and they include: transcription factors (TF), kinases, phosphatases, among others. The main characterized TF families regulating abiotic stress responses in plants include: AP2/ERF (APETALA2/ethylene-responsive element-binding factor), DREB (dehydration-responsive element-binding), bZIP (basisc leucine zipper), AREB/ABF (ABA-responsive element-binding protein/ABA-binding factor), NAC (NAM, ATAF1/2, CUC), MYB (myeloblastosis oncogene), bHLH (basic helix-loop-helix proteins), and WRKY^10–14^.

Efficient communication of detected external signals between distal tissues is of great importance for coordinated plant development and to generate rapid responses against unfavorable conditions. The vascular system regulates the long-distance trafficking of several molecules (water, nutrients, photoassimilates, among others) between distal tissues^15^. The phloem sap is responsible for the movement, distribution and trafficking of these and other macromolecules^16^, such as proteins and RNAs. However, the molecular participation of the phloem in response to drought is less studied.

Due to the complex molecular responses of plants to drought and other types of stress, omics approaches have been implemented to unravel their intricate mechanisms^17,18^. The development of Next Generation Sequencing (NGS) technologies, or High-Throughput Sequencing, has permitted the analysis of several plants transcriptomes through the sequencing of their RNA (RNA-Seq) species, e.g mRNA, microRNA, etc.^19–21^. This has generated substantial data sets of hundreds or thousands of regulated genes in response to drought. Bioinformatic tools have been developed to analyze the enormous quantity of information generated, such as enrichment analyses, to determine meaningful regulated biological processes^22^. Also, co-expression networks have been applied for the identification of putative regulatory transcriptional hubs in plants^23,24^. These advances in technology and *in silico* analysis have permitted the elucidation of molecular mechanisms in model and non-model plants, including several economically important agricultural crops.

Papaya plant (*Carica papaya* L.) is a fruit crop grown world-wide in tropical and sub-tropical regions. Papaya fruit is a rich source of nutrients and papain, a digestive enzyme with several industrial applications. In 2016, Mexico was the third largest producer of papaya fruit, having produced about 951,922 metric tons^25^. Additionally, Mexico has been the leading exporter worldwide for several years^25^. Papaya plants have been considered relatively resistant to drought, responding through a desiccation postponement mechanism^26^. Upon drought stress papaya plants accumulate proline^27^ and ions, such as K^+^, Na^+^, and Cl^−2^ probably contributing to osmotic adjustment. Levels of ABA and Jasmonic acid (JA) hormones have also been found to increase in response to drought in papaya^27,28^. However, water scarcity may limit papaya physiological performance^26–28^. Previous studies on papaya transcriptomes have focused on the analysis of root specific gene expression^29^, fruit ripening^30^, sex determination^31^, cold-induced sex reversal^32^, expression changes in the papaya ringspot virus (PRSV)-resistant transgenic ‘Sunup’^33^, somatic embryogenesis^34^, and sticky disease responses^35^. However, transcriptomic analyses for the elucidation of papaya plant molecular responses to drought remain scarce.

In the present study we employed Illumina RNA-Seq to analyze the transcriptome of leaves, sap, and roots of papaya plants under well-watered (control) condition, and after 10 and 20 days of drought stress. We identified tissue-specific sets of differentially expressed genes (DEGs) through reference and *de novo* assembly approaches. Functional enrichment analysis of these sets of DEGs revealed specific biological processes regulated among tissues under the control condition, and in response to drought. Furthermore, through co-expression network analysis, we identified several abiotic stress related TFs, which may act as putative regulatory hubs in leaves and roots under drought stress. Our findings provide a profound understanding of the molecular responses of papaya plant to drought stress, and provide critical baseline information for future genetic improvement and breeding programs of this important tropical fruit crop.

## Results

### Effect of drought stress on papaya plants

**Figure 1:**
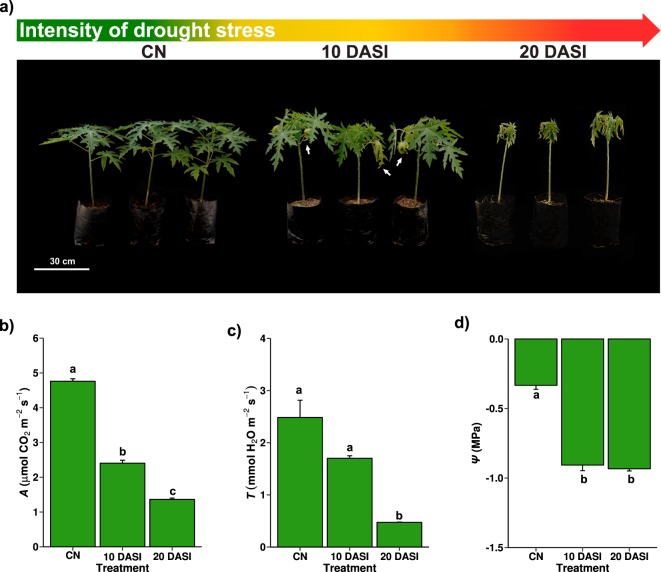
Effect of drought stress on papaya plants. (**a**) Three-month-old ‘Maradol’ papaya plants under control condition (CN) and at 10 and 20 days after stress imposition (DASI). The gradient-colored arrow indicates the intensity of the imposed stress: green = well-watered or no stress, yellow = mild stress, and red = severe stress. White arrows in the plants at 10 DASI indicate leaf wilting. (**b**–**d**) Effect of drought stress on papaya plant physiology: (**b**) CO2 assimilation rate (*A*), (**c**) transpiration rate (*T*), and (**d**) leaf water potential (*Ψ*) of the papaya plant depicted in a). Bars and error bars represent means and standard deviation (s.d.), respectively, of three independent plants (n = 3). Different letters indicate statistically significant differences (ANOVA, Tukey p < 0.001).

We imposed drought stress by stopping watering on three-month-old ‘Maradol’ papaya plants. Visual examination of the plant phenotypes and physiological measurements were performed under control (CN) condition, and at 10 and 20 days after stress imposition (DASI). Under CN condition the plants appeared healthy and presented dark-green colored leaves (Fig. 1a). At 10 DASI the plants still retained most of their leaves, however some leaves were curled and wilted (indicated with white arrows in Fig. 1a). At 20 DASI the plants had lost most of their leaves and the remaining leaves were very curled and wilted (Fig. 1a). The CO2 assimilation rate (*A*), transpiration rate (*T*), and leaf water potential (*Ψ*) of the plants under CN condition presented mean values of 4.76 ± 0.07 s.d. µmol CO^2^ m^−2s−1^, 2.48 ± 0.33 s.d. mmol H2O m^−2s−1^, and −0.33 ± 0.02 s.d. MPa, respectively (Fig. 1b–d). Compared to CN plants, *A* mean value of the drought-stressed plants significantly decreased (Tukey p < 0.001) by 49.5 and 71.3% at 10 and 20 DASI, respectively (Fig. 1b). *T* mean value decreased by 31.5% at 10 DASI, but was significantly reduced (Tukey p < 0.001) by 80.9% until 20 DASI (Fig. 1c)*Ψ* mean value significantly decreased (Tukey p < 0.001) by 172 and 180% at 10 and 20 DASI, respectively (Fig. 1d). We isolated RNA samples from leaves (L), sap (S), and roots (R) of these same plants and performed both reference and *de novo* RNA-seq transcriptomic analyses.

### RNA-Sequencing and expression level quantification

**Figure 2:**
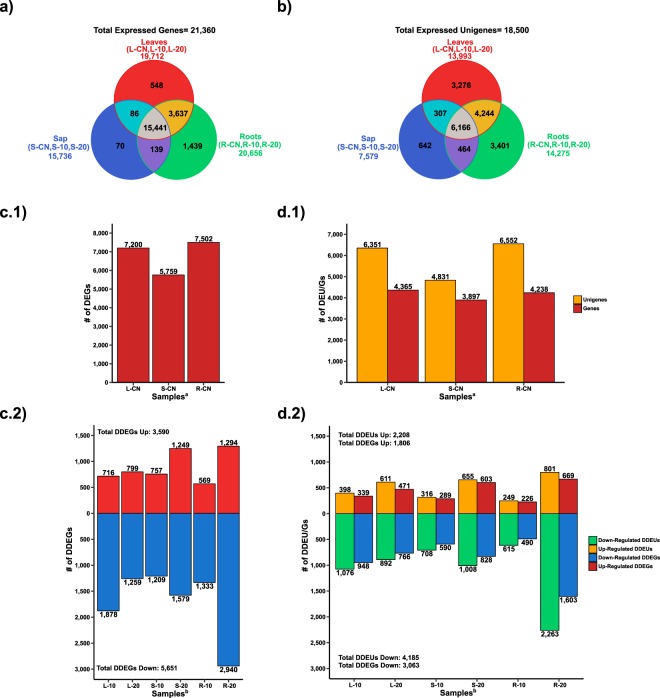
Reference-based and *de novo* transcriptomic analyses. (**a**) Total number of expressed genes (TPM ≥ 1) per tissue identified in the reference-based transcriptomic approach. (**b**) Total number of expressed unigenes (TPM ≥ 16) per tissue identified in the *de novo* transcriptomic approach. (**c.1**) Number of up-regulated genes among tissues under control (CN) condition identified in the reference-based transcriptomic analysis. (**d.1**) Number of up-regulated unigenes (yellow) and corresponding gene models (red) among tissues under CN condition identified in the *de novo* transcriptomic analysis. (**c.2**.) Number of up-regulated (red) and down-regulated (blue) DDEGs in stressed tissues identified in the reference-based transcriptomic analysis. (**d**.**2**) Number of up-regulated DDEUs (yellow) and corresponding gene models (red), and down-regulated DDEUs (turquoise) and corresponding gene models (blue) in stressed tissues identified in the *de novo* transcriptomic analysis. DEU/G = Differentially expressed unigenes/genes; DDEU/G = Drought-responsive differentially expressed unigenes/genes. Sample names are described in Table 1. ^a^Comparisons to obtain the number of up-regulated genes by tissue type under control condition: L-CN = L-CN_vs_S-CN + L-CN_vs_R-CN; S-CN = S-CN_vs_L-CN + S-CN_vs_R-CN; R-CN = R-CN_vs_L-CN + R-CN_vs_S-CN. ^b^Comparisons to obtain the number of up- and down-regulated genes under drought stress: L-10 = L-CN_vs_L-10; L-20 = L-CN_vs_L-20; S-10 = S-CN_vs_S-10; S-20 = S-CN_vs_S-20; R-10 = R-CN_vs_R-10; R-20 = R-CN_vs_R-20.

We sequenced a total of 18 cDNA libraries from 9 samples (in duplicates): S-CN, R-CN, L-CN, S-10, R-10, L-10, S-20, R-20, and L-20 (Table 1). We obtained a total of 621,077,480 raw reads, but we only kept 617,929,151 (99.49%) clean reads (Q > 30) (Table 1), which were used to *de novo* assemble the papaya transcriptome by means of Trinity software^36^ (for details please see Supplementary Fig. S1 and Table S1). Expression levels were quantified by mapping the clean reads of each sample to both the extant reference genome of the transgenic ‘SunUp’ papaya^37^ and to our *de novo* assembled transcriptome. Hereafter, we will refer to as “genes” to the gene models from the reference genome, and “unigenes” to the features assembled in our *de novo* transcriptome. We only considered genes and unigenes as “expressed” if they had TPM values ≥1 and ≥16, respectively. Based on this delimitation, a total of 21,360 genes (77% of the total 27,699 genes present in the ‘SunUp’ papaya genome) were expressed in the reference-based transcriptomic analysis (Fig. 2a and Supplementary Table S2). Conversely, a total of 18,500 unigenes were expressed (TPM ≥ 16) in our *de novo* transcriptome assembly (Fig. 2b and Supplementary Table S3), and of those, 11,864 unigenes represented 43% of the total gene models from the reference genome (27,699 genes). In both reference and *de novo* transcriptomic analyses, Pearson’s correlation coefficient based on expression values of each library indicated high correlation among sample replicates (Supplementary Fig. 2a,b). Hierarchical clustering and Principal Component Analysis (PCA) indicated major grouping of the samples according to tissue type, rather than stress treatment (Supplementary Fig. 2a–d). We performed differential expression analysis on the sets of 21,360 and 18,500 expressed genes and unigenes.

**Table 1:** Samples and libraries used for the *de novo* and reference-based transcriptomic analyses.

Sample^a^	Treatment^b^	Library^c^	Raw Reads	GC%	Clean Reads (Q > 30)	GC%
S-CN	Control	S-CNa	27,805,197	42	27,640,601	42
		S-CNb	37,912,534	43	37,736,959	43
R-CN	Control	R-CNa	47,387,690	44	47,091,639	43
		R-CNb	36,666,468	43	36,503,924	43
L-CN	Control	L-CNa	42,292,905	44	42,143,433	44
		L-CNb	43,896,258	44	43,743,975	44
S-10	10 DASI	S-10a	32,528,720	43	32,077,543	43
		S-10b	39,861,192	44	39,687,165	44
R-10	10 DASI	R-10a	32,723,799	45	32,550,260	45
		R-10b	31,347,259	44	31,214,117	44
L-10	10 DASI	L-10a	22,994,548	44	22,902,184	44
		L-10b	39,835,409	44	39,702,331	43
S-20	20 DASI	S-20a	30,694,925	43	30,495,357	43
		S-20b	31,872,909	43	31,711,157	42
R-20	20 DASI	R-20a	33,133,317	43	32,986,618	43
		R-20b	26,405,792	44	26,292,893	43
L-20	20 DASI	L-20a	35,290,183	43	35,144,767	43
		L-20b	28,428,375	44	28,304,228	43
Totals=			**621,077,480**		**617,929,151**	

^a^L = leaves, S = sap, R = roots; CN = Control, 10 = 10 DASI, 20 = 20 DASI.

^b^DASI = days after stress imposition.

^c^“a” and “b” indicate sample replicates.

### Differential Expression Analysis

In the reference-based transcriptomic analysis, under control condition we found totals of 7,200, 5,759, and 7,502 up-regulated genes in L-CN, S-CN, and R-CN, respectively (Fig. 2c.1, Supplementary Fig. S3, and Supplementary Table S4). Moreover, under drought-stress conditions we identified a total of 8,549 Drought-responsive Differentially Expressed Genes (DDEGs) of which 3,590 were up-regulated and 5,651 were down-regulated (Fig. 2c.2 Supplementary Fig. S4, and Supplementary Table S4). Hierarchical clustering of the expression values of the total DDEGs across samples revealed 7 major clusters; the samples were grouped, firstly, according to tissue type, and secondly, according to stress treatments (Supplementary Fig. S5a,b).

In the *de novo* transcriptomic analysis, under CN condition we found totals of 6,351, 4,831, and 6,552 up-regulated unigenes in L-CN, S-CN, and R-CN, respectively (Fig. 2d.1, Supplementary Fig. S6, and Supplementary Table S5). These unigenes corresponded to 4,365, 3,897, and 4,238 gene models from the reference genome in L-CN, S-CN, and R-CN, respectively (Fig. 2d.1). Moreover, under drought-stress conditions we found a total of 6,089 Drought-responsive Differentially Expressed Unigenes (DDEUs) of which 2,208 were up-regulated and 4,185 were down-regulated (Fig. 2d.2, Supplementary Fig. S7, and Supplementary Table S5). These unigenes corresponded to 1,806 up-regulated gene models, and 3,063 down-regulated gene models, from the reference genome of the transgenic ‘SunUp’ papaya (Fig. 2d.2). Clustering analysis of the total DDEUs revealed 6 major expression patters; the samples were grouped, firstly, according to tissue type, and secondly, according to stress treatments (Supplementary Fig. S5c,d).

Between 58 and 85% of the DDEUs identified by means of the *de novo* approach, were shared with the DDEGs identified in the reference-based approach (Supplementary Fig. S8a–c). This indicates that both transcriptomic approaches recovered similar sets of differentially expressed gene models from the reference genome. We only utilized the genes from the reference-based transcriptomic analysis for further enrichment and co-expression analyses.

### Core sets of DDEGs identified in the reference-based transcriptomic analysis

**Figure 3:**
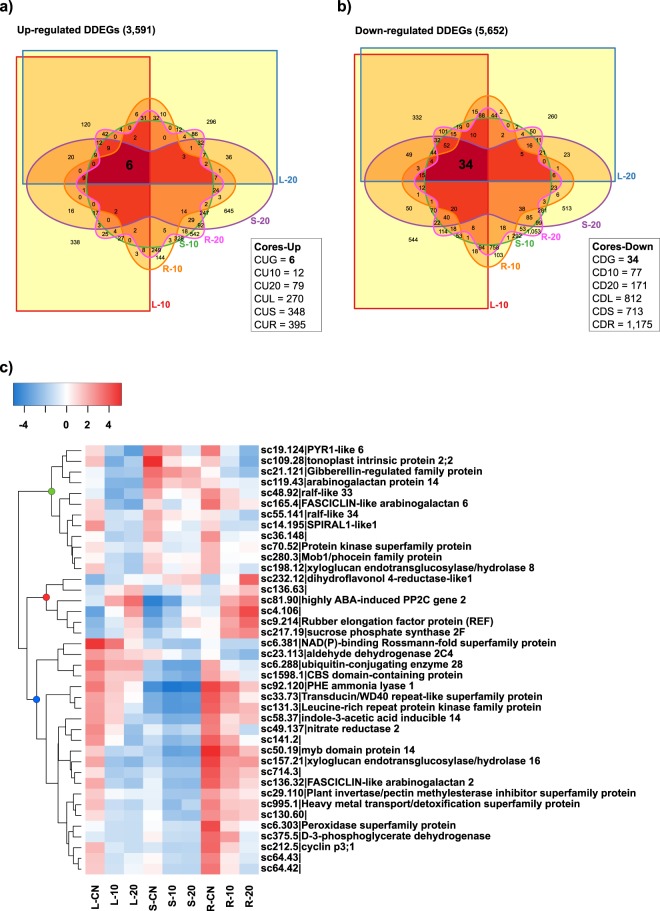
Shared core sets of up- and down- regulated DDEGs identified in the reference-based transcriptomic analysis. (**a**) Venn diagram depicting the intersections of all sets of up-regulated DDEGs. (**b**) Venn diagram depicting the intersections of all sets of down-regulated DDEGs. **c)** Heatmap depicting the expression of the shared core sets of up- or down-regulated DDEGs (CUG and CDG respectively) in all tissues and treatments. In a) and (**b**): CUG = core of up-regulated genes in all tissues during stress treatments; CU10 = core of up-regulated genes in all tissues at 10 DASI; CU20 = core of up-regulated genes in all tissues at 20 DASI; CUL = core of up-regulated genes in L-10 and L-20; CUS = core of up-regulated genes in S-10 and S-20; CUR = core of up-regulated genes in R-10 and R-20; CDG = core of down-regulated genes in all tissues during stress treatments; CD10 = core of down-regulated genes in all tissues at 10 DASI; CD20 = core of down-regulated genes in all tissues at 20 DASI; CDL = core of down-regulated genes in L-10 and L-20; CDS = core of down-regulated genes in S-10 and S-20; CDR = core of down-regulated genes in R-10 and R-20. In (**c**) green circle = clade of genes relatively highly expressed under CN condition in leaves, sap, and roots; red circle = clade of the core set of 6 up-regulated DDEGs in all tissues and treatments (CUG); blue circle = clade of genes relatively highly expressed in leaves and roots, but lowly expressed in sap, under CN condition. In gene annotations in (**c**): “sc” = “evm.TU.supercontig_”. Heatmap color key indicates the mean-centered log2(TPM + 1) values of the mean of TPMs per sample duplicates. DDEG = Drought-responsive differentially expressed genes. Separate shared core sets are depicted in Supplementary Fig. S9. Samples names are described in Table 1.

We compared the intersection of all sets of up- and down-regulated DDEGs (identified in our reference-based transcriptomic analysis) across all tissues under stress treatments to determine shared core sets. We found 6 genes that were inside of a shared Core set of up-regulated genes (CUG) (Fig. 3a) and 34 genes that were inside a shared Core set of down-regulated genes (CDG), across all samples under stress treatments (Fig. 3b). The CUG set included evm.TU.model_supercontig_232.12 (dihydroflavonol 4-reducatse-like1), evm.TU.model_supercontig_81.90 (highly ABA-induced PP2C gene 2), evm.TU.model_supercontig_9.214 (Rubber elongation factor protein (REF)), and evm.TU.model_supercontig_217.19 (sucrose phosphate synthase 2 F) (clade marked with a red circle in Fig. 3c). Additionally, twelve genes within the CDG set had relatively high expression in leaves, sap, and roots under CN condition (clade marked with a green circle in Fig. 3c). These genes included evm.TU.model_supercontig_19.124 (PYR1-like 6), evm.TU.model_supercontig_109.28 (tonotoplast intrinsic protein 2;2), and evm.TU.model_supercontig_21.12 (Gibberellin-regulated family protein) (Fig. 3c). Twenty-two genes within the CDG set had relatively high expression in roots and leaves, but not sap, under CN condition (clade marked with a blue circle in Fig. 3c). These genes included evm.TU.model_supercontig_50.19 (myb domain protein 14), evm.TU.model_supercontig_131.3 (Leucin-rich repeat protein kinase family protein), and evm.TU.model_supercontig_6.303 (Peroxidase superfamily protein) (Fig. 3c). Other core sets of DDEGs were identified for 10 DASI and 20 DASI treatments for each plant tissue. For example, 270 (CUL), 368 (CUS), and 395 (CUR) DDEGs were determined as up-regulated cores; and 812 (CDL), 348 (CDS), and 1,175 (CDR) DDEGs were determined as down-regulated cores in leaves, sap, and roots, respectively (Fig. 3a,b, Supplementary Fig. S9a, and Supplementary Table S2). Twelve (CU10) and 79 (CU20) DDEGs were up-regulated in all three tissues, and 77 (CD10) and 171 (CD20) DDEGs were down-regulated in all three tissues, at 10 DASI and 20 DASI, respectively (Fig. 3a,b, Supplementary Fig. S9b, and Supplementary Table S2).

### GO functional enrichment analysis of the sets of differentially expressed genes under control condition

**Figure 4:**
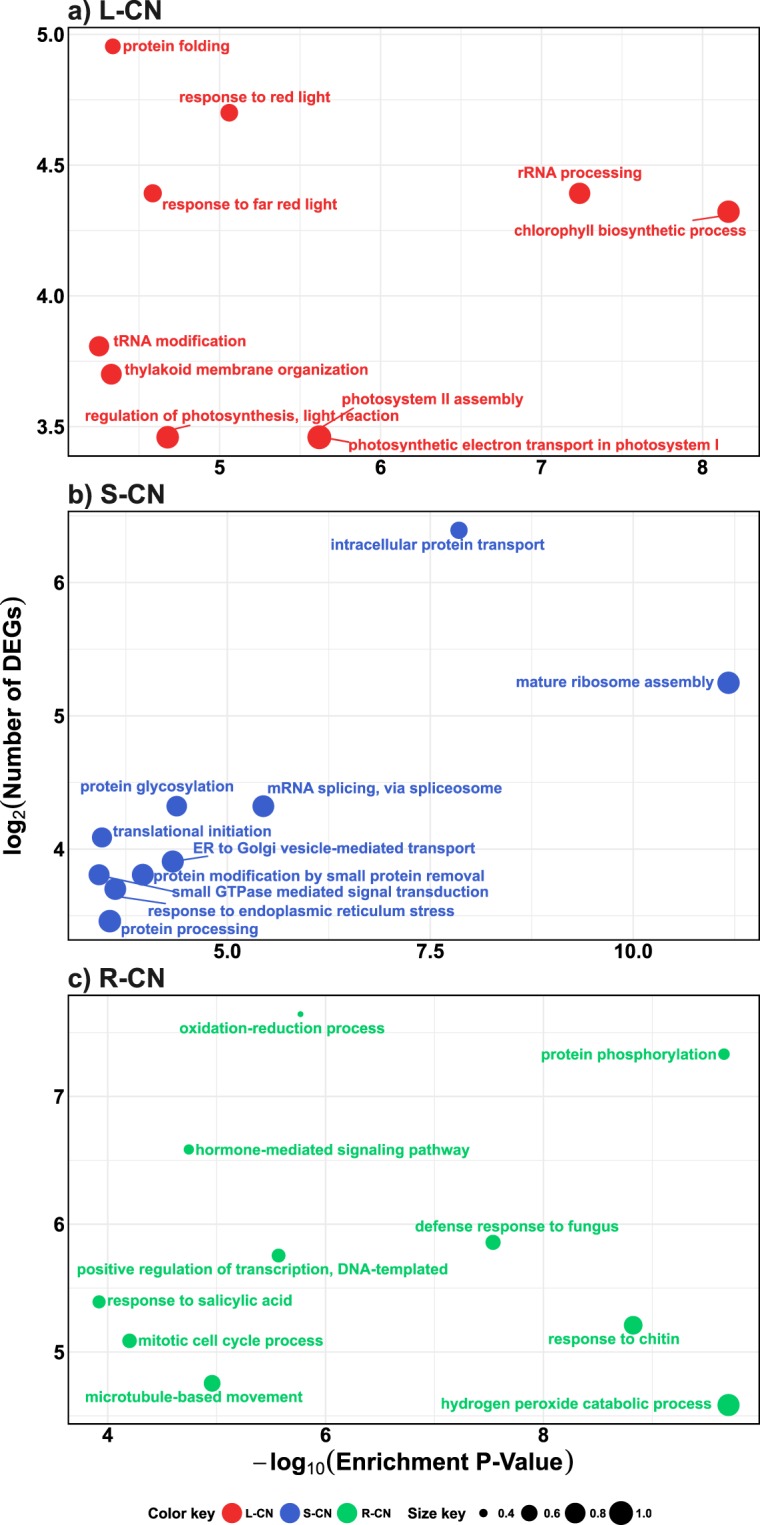
Functional GO enrichment analysis of the sets of up-regulated genes in leaf, sap, and roots under control condition identified in the reference-based transcriptomic analysis. Top 10 enriched GO terms (Biological Process category) identified in (**a**) L-CN, (**b**) S-CN, and (**c**) R-CN. Only the top 10 (based on enrichment P-Value) enriched GO terms are plotted in function of their enrichment P-value (x axis) and number of genes (y axis). Size key indicates the ratio of the number of genes in the test-set/total number of genes, for any given GO term. The complete lists of enriched GO terms are presented in Supplementary Table S6.

We identified enriched GO terms in tissue-specific sets of DEGs under CN condition identified by our reference-based transcriptomic approach, i.e. 2,641, 3,527, and 2,703 DEGs for L-CN, S-CN, and R-CN, respectively **(**Supplementary Fig. S3d). Totals of 35, 13, and 36 enriched GO terms were found in L-CN, S-CN, and R-CN, respectively (Supplementary Table S6). As expected, in L-CN several enriched GO terms (17) were related to photosynthesis and response to light, for example: “chlorophyll biosynthetic process”, “photosynthetic electron transport in photosystem I”, “photosystem II assembly”, and “response to red light” (Fig. 4a, and Supplementary Table S6). Other GO terms were found that related to development (ovule development), rRNA and tRNA metabolism, pigment biosynthesis (chlorophyll), and seven biosynthetic pathways, among other biological processes (Supplementary Fig. S10a, and Supplementary Table S6). The S-CN sample was enriched in GO terms related to transport (“intracellular protein transport”, “ER to Golgi vesicle-mediated transport”, “vacuolar transport”), RNA and protein modification (“mRNA splicing, via spliceosome”, “protein glycosylation”, “protein modification by small protein removal”, “proteasome-mediated ubiquitin-dependent protein catabolic process”, “protein processing”), translation (“mature ribosome assembly”, “translational initiation”) and stress response (“response to endoplasmic reticulum stress”), among others (“small GTPase mediated signal transduction” and “vacuole organization”) (Fig. 4b, Supplementary Fig. S10b, and Supplementary Table S6). In R-CN the 10 most enriched GO terms were: “hydrogen peroxide catabolic process”, “protein phosphorylation”, “response to chitin”, “defense response to fungus”, “oxidation-reduction process”, “positive regulation of transcription, DNA-templated”, “microtubule-based movement”, “hormone-mediated signaling pathway”, “mitotic cell cycle process”, and “response to salicylic acid” (Fig. 4c, and Supplementary Table S6). Other GO terms related to the regulation of cellular process, cell wall metabolism, development, and secondary metabolism were also represented (Supplementary Fig. S10c, and Supplementary Table S6).

### GO functional enrichment analysis of the sets of DDEGs

**Figure 5:**
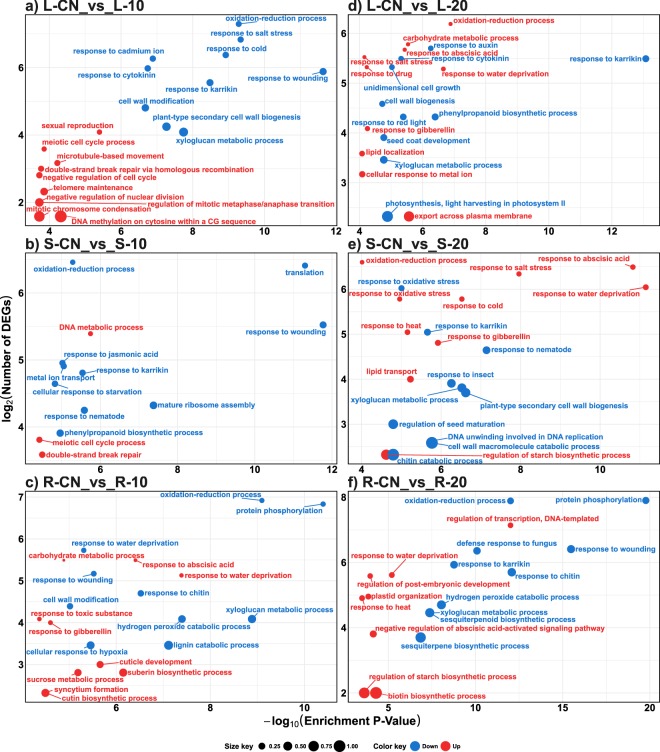
Functional GO enrichment analysis of the sets of DDEGs identified in the reference-based transcriptomic analysis. Top 10 enriched GO terms (Biological Process category) identified in the sets of up-regulated (red) and down-regulated (blue) DDEGs in (**a**) L-CN_vs_L-10, (**b**) S-CN_vs_S-10, (**c**) R-CN_vs_R-10, (**d**) L-CN_vs_L-20, (**e**) S-CN_vs_S-20, and (**f**) R-CN_vs_R-20. Only the top 10 (based on enrichment P-Value) enriched GO terms are plotted in function of their enrichment P-value (x axis) and number of genes (y axis). GO terms in sets of up-regulated and down-regulated genes are indicated in red and blue, respectively. Size key indicates the ratio of the number of genes in the test-set/total number of genes, for any given GO term. DDEG = Drought-responsive differentially expressed genes. The complete lists of enriched GO terms are presented in Supplementary Table S6.

We found enriched GO terms for each tissue (leaf, sap, and root) at a specific time-point stress treatment (10 and 20 DASI). All sets of up-regulated and down-regulated GO terms can be inspected in the Supplementary Table S6. At 10 DASI, five of the top 10 enriched GO terms in the up-regulated gene set in leaves (L-10) were related to cell cycle, three were related to DNA molecule, one to sexual reproduction, and one to micro-tubule based movement. (Fig. 5a, Supplementary Fig. S11a and Supplementary Table S6). In sap (S-10) “DNA metabolic process”, “double-strand break repair”, and “meiotic cell cycle process” were enriched in the up-regulated gene set (Fig. 5b, Supplementary Fig. S11b, and Supplementary Table S6). In roots (R-10), four terms related to cuticle or suberin formation, two to abiotic stress response (water and salt), two to hormones (ABA and gibberellin), two to carbohydrate metabolism, among others, were enriched in the up-regulated gene set (Fig. 5c, Supplementary Fig. S11c, and Supplementary Table S6). At 20 DASI response to water deprivation, ABA-related responses, and carbohydrate metabolism were enriched in the up-regulated gene sets of all three samples (L-20, S-20, and R-20). Other GO terms related to heat, salt, and oxidative stresses were also enriched in the up-regulated gene sets at 20 DASI (Fig. 5d–f, Supplementary Fig. S11d–f, and Supplementary Table S6). At both 10 and 20 DASI (and in all tissues), we found enriched GO terms in the down-regulated gene sets that related to: abiotic stress responses (such as water and osmotic stresses), defense related hormones (such as jasmonic acid, salicylic acid, and abscisic acid), defense against biotic stresses, cell wall metabolism, oxidation-reduction, ion transport, and development (Fig. 5a–f, Supplementary Fig. S11g–l, and Supplementary Table S6). “Response to karrikin” was also an enriched GO term in all down-regulated sets of DDEGs (Fig. 5a–f, and Supplementary Table S6).

### Co-expression networks

**Figure 6:**
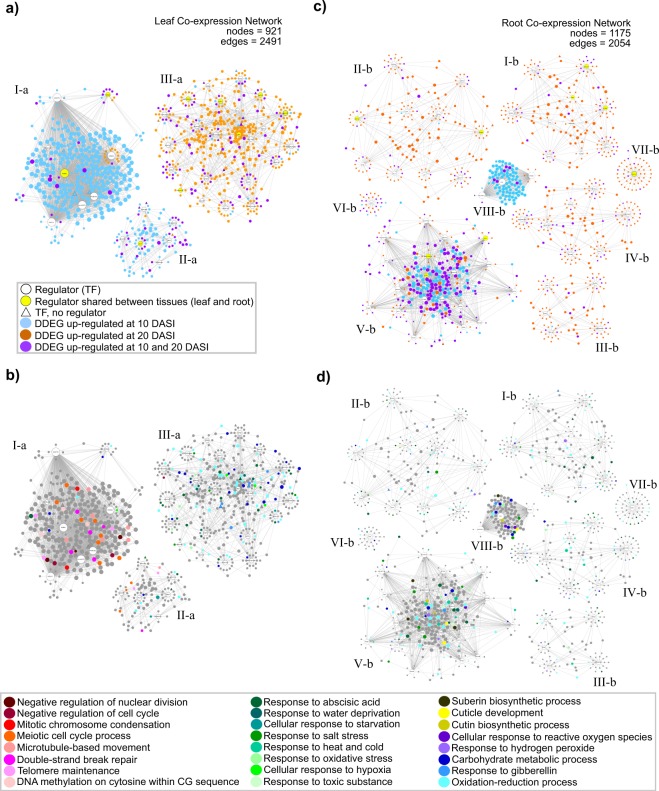
Gene co-expression networks (GCN) of leaf and root transcriptomes of papaya plant during drought treatments. Up-regulated TFs, previously reported in response to abiotic stress and found in the stress datasets were set as “Regulators” for the construction of the networks. (**a**) GCN for leaf tissues showing time-point stress responses of up-regulated DDEGs. The three communities are labeled as I-a, II-a, and III-a, and the 37 most connected TFs are depicted in the network as white circles. (**b**) Same GCN as in a), but showing the location in the network of genes belonging to different enriched GO groups by Biological Process (BP). (**c**) GCN for root tissues showing time-point stress responses of up-regulated DDEGs. The eight communities are labeled as I-b–VIII-b, and the 56 most connected TFs are depicted in the network as white circles. (**d**) Same GCN as in b), but showing the location in the network of genes belonging to different enriched GO groups by BP. Nodes in (**a**) and (**c**) are colored according the time-point stress treatments, and nodes in (**b**) and (**d**) are colored according to enriched GO groups as shown in the Fig. legends. DASI = days after stress imposition. Detailed networks are depicted in Supplementary Figs S12 and S13.

Based on TPM values of up-regulated DDEGs only, we built two independent gene co-expression networks (GCNs), one for leaves and another for roots, and detected natural gene co-expression communities (Fig. 6 and Supplementary Figs S12 and S13). In such networks the up-regulated DDEGs are represented by nodes, and pairwise co-expression relationships between DDEGs are represented by edges. Selected TFs were set as “regulator nodes” of the network in order to detect regulatory hubs (listed in the “Regulator nodes” column in Supplementary Tables S7 and S8). For the leaf GCN we determined three communities (I-a–III-a) composed of a total of 921 DDEGs (nodes) connected to 37 (of 39) TFs set as “regulator nodes” (Fig. 6a and Supplementary Table S7). Moreover, 42.9, 39.8 and 17.1% of these 921 DDEGs were up-regulated at 10 DASI, at 20 DASI, and at both stress treatments, respectively (see node color key in Fig. 6a). These DDEGs were clearly clustered according to time-point treatments: Communities I-a and II-a presented a high proportion (88 and 69% respectively) of up-regulated DDEGs at 10 DASI (blue nodes); community III-a presented a high proportion (74%) of up-regulated DDEGs at 20 DASI (orange nodes) and contained most of the genes (10.9%) that were up-regulated under both stress treatments in the leaf GNC (purple nodes, Fig. 6a). Furthermore, 69 genes belonging to enriched GO terms related to cell cycle process (nodes in different red tones, orange, and pink) were clustered at 10 DASI in the I-a community (Fig. 6b), and 36, 44, and 22genes belonging to enriched GO terms related to abiotic stress stimulus (nodes in different green tones), oxidation-reduction (cyan nodes), and carbohydrate metabolic processes (dark blue nodes) were clustered at 20 DASI in the III-a community (Fig. 6b).

For the root GCN we determined eight communities (I-b–VIII-b) composed of a total of 1,175 DDEGs (nodes) connected to the total 56 TFs set as “regulator nodes” (Fig. 6c and Supplementary Table S8). In this root GCN the nodes (DDEGs) were more scattered and less connected than in leaf GCN (compare Fig. 6a,c). Different from the leaf GCN, in the root GCN most of the DDEGs (59.3%) were up-regulated at 20 DASI, followed by those shared (elements) by both stress treatments (26%), and finally those DDEGs up-regulated at 10 DASI (14.6%) (see node color key in Fig. 6c). Five communities (I-b–IV-b, and VII-b) contained a high proportion (between 79 and 88% per community) of up-regulated DDEGs at 20 DASI (orange nodes) and a low proportion (between 11 and 19%) of shared elements (purple circles). Community V-b was composed by a mix DDEGS up-regulated at 10 or 20 DASI (24 and 25% respectively; blue and orange nodes) and a high percentage (50.1%) of shared elements between both stress treatments (purple circles). Community VI-b contained high proportions of DDEGs up-regulated at 20 DASI (40%) and under both stress treatments (43%), and a low proportion of DDEGs at 10 DASI (16%). Community VIII-b consisted primarily (84%) of DDEGs up-regulated at 10 DASI (blue nodes) (Fig. 6c). Furthermore, we found that the enriched GO terms related to cellular responses, to abiotic stress stimulus (nodes in different green tones), oxidation-reduction process (cyan nodes), responses to reactive oxygen species (purple nodes), and cutin and cuticle development (yellow nodes) were mainly found in communities V-b and VIII-b from the 10 DASI (Fig. 6d). The remaining communities showed a minimal and scattered distribution of GO terms.

### TFs as regulatory hubs

In both leaf and root GCNs, we identified the regulator nodes (i.e. selected TFs) with highest connectivity because of their relevance as regulatory hubs in response to drought (highlighted in red in the Degree column in Supplementary Tables S7 and S8). In the leaf GCN, the six TFs with the highest degree distribution (from 299 to 266) were WRKY70 (evm.TU.supercontig_19.44), MYB94 (evm.TU.supercontig_111.6), RAP2.11 (evm.TU.supercontig_51.136), bHLH (STP; evm.TU.supercontig_55.132), HSFB-2A (evm.TU.supercontig_107.31), and AP2/ERF (evm.TU.supercontig_2.268) homologues (Supplementary Table S7). These six TFs were clustered in the community I-a and linked with other DDEGs at 10 DASI (Supplementary Fig. S12). In contrast to the leaf GNC, in the root GNC the TFs regulators were less connected and with lower degree distribution values. The three regulators in the roots with the highest degree distribution (from 91 to 86) were MYB63 (evm.TU.supercontig_34.3), bHLH (ICE1; evm.TU.supercontig_70.77), and bHLH (evm.TU.contig_26556.1) homologues (Supplementary Table S8). These three regulators were clustered together in the community VIII-b, composed mainly of genes up-regulated at 10 DASI (Supplementary Fig. S13). Furthermore, nine TFs regulators were shared between the leaf and root GCNs, which are homologues of ABI5 (evm.TU.supercontig_5.28), RAP2.6 (evm.TU.supercontig_38.79), bHLH (evm.TU.supercontig_20.63), ANAC072 (RD26; evm.TU.supercontig_80.93), ANAC074 (evm.TU.supercontig_165.12), MYB48 (evm.TU.supercontig_190.35), MYB94 (evm.TU.supercontig_111.6), bZIP1 (evm.TU.supercontig_9.75), and WRKY75 (evm.TU.supercontig_807.4) (yellow nodes in Fig. 6a,b, Supplementary Fig. S12 and S13, and Supplementary Tables S7 and S8***)****.* In the leaf GCN, six of these regulators were clustered at 20 DASI in the III-a community; only MYB94 was among the highest degree distribution TFs mentioned above (Supplementary Fig. S12). In the root GCN, the nine TFs were included in the I-b, II-b, V-b, and VII-b communities (Supplementary Fig. S13); however, none of them was among the highest degree distribution TFs grouped in community VIII-b.

## Discussion

### Biological processes differentially modulated under CN condition

Papaya tissues clearly presented different expression patterns (Supplementary Figs S2 and S5). Under the control condition, root tissues were already enriched with GO terms that might relate to stress response and defense, such as hydrogen peroxide catabolic process (Fig. 4c). Similar results were obtained from the transcriptomic analysis of papaya roots in a previous study^29^. Evidently, the roots maintain these processes for a rapid response upon water deficit. The sap tissue presented a unique transcriptomic profile enriched with several biological processes related to mRNA splicing via spliceosome, RNA and protein transport, protein post-translational modifications (for example glycosylation, deneddylation, and proteolysis), regulation of localization, SNARE interactions, and stress (even under CN condition) (Supplementary Fig. S10b). Similar results were identified in the transcriptome of sap in melon fruit^38^. Grafting experiments on papaya plants could provide a further understanding of how transcripts move long-distance through the phloem sap. The transcriptome profile in leaves was enriched with GO terms related to photosynthesis and chloroplast metabolic processes as expected (Fig. 4a). Promoter regions of these tissue-specific genes (from leaves, sap, and roots) could be useful for the regulation of cis-genes in papaya plant.

### Shared core sets of stress regulated genes, and genes related to water deprivation and ABA as targets for genetic improvement in papaya

We found shared core sets of 6 and 34 DDEGs that were up- or down-regulated, respectively, in all stressed samples (Fig. 3a,b). Interestingly, in the shared core set of up-regulated genes, the Rubber elongation factor protein (REF) showed an important transcriptional regulation in the three studied tissues (Fig. 3c). This protein takes part in the biosynthesis of natural rubber (a component of latex) which has been implied as playing a key role in defense against pathogens, preventing their entry into wounded tissues^39^. This finding is interesting because papaya is a lactiferous species that shows a dense network of articulated and anastomosing laticifer vessels in leaves, stems^39^ and root tissues^40^. Homologs of REF protein in non-rubber producing species such as hot pepper demonstrate its involvement in drought tolerance and other stress conditions^41,42^. So, this drought responsive REF homolog in papaya could also be involved in mechanisms that both prevent pathogen attacks under unfavorable conditions and cope with abiotic stress. Together with other candidate genes, these sets of shared core genes are promising targets for further functional studies addressing genetic improvement due to their consistent response and expression across all samples. On the other hand, we found 142 and 192 only-up- and only-down-regulated genes under any treatment, which were enriched in terms related to water deprivation or ABA (Supplementary Fig. S14 and Table S2). These gene sets included several genes involved in ABA metabolism and signaling in response to stress, such as ABI1, ABI5, PYR1-like, and Protein phosphatase 2 C (Supplementary Fig. S14a,b). We also found several TFs related to MYB, HD, bZIP, bHLH, NAC, and WRKY families; chaperones; and redox-related enzymes. The functional analysis of these genes *in planta* could lead to the development of crop varieties with higher tolerance to drought. For example, TFs are attractive targets for the generation of plants with higher tolerance to abiotic stresses^43^, since TFs are able to simultaneously control the expression of many stress related genes.

### Biological processes differentially up-regulated under moderate drought stress

At 10 DASI the papaya plants were already under the effects of the imposed drought stress as demonstrated by the reduction in physiological performance (Fig. 1a); however, the plants still retained several leaves, so we designated this time-point as a moderate drought stress. Like other types of stress, drought increases the production of ROS, which can damage different types of molecules, including DNA. This damage can result in reduced protein synthesis and genomic instability, which can ultimately contribute to a reduction in plant performance^44^. In the present study we found that double-strand break-repair processes were enriched in the up-regulated gene sets in leaves and sap, under moderate drought stress condition (10 DASI) (Fig. 5a,b). In both, leaf and sap, homologues of RPA32B (evm.TU.supercontig_233.2) and RAD54 (evm.TU.supercontig_62.19) were up-regulated (Supplementary Table S4). Moreover, in roots we found enriched GO terms related to common abiotic stress responses such as water deprivation, salt stress, ABA, and oxidation-reduction process (Supplementary Table S6). We also found that genes related to suberin biosynthesis were enriched in roots at 10 DASI. Suberin is an extracellular biopolymer found in the cell walls of aerial and underground tissues of plants, which modulates water movement and solute uptake, and is thought to play an important role in plant tolerance against drought, salinity, and pathogen attack^8,45^. In this study several genes involved in the synthesis and transport of suberin monomers were up-regulated in roots at 10 DASI, such as homologues of 3-ketoacyl-CoA synthase 2 (evm.TU.supercontig_1103.1), cytochrome P450 (evm.TU.supercontig_112.48), and ABC-2 type transporter (evm.TU.supercontig_114.19). These results suggest that under moderate drought stress papaya plants preferentially regulate different biological processes in roots, as opposed to the sap and leaves, conceivably because roots experience drought stress first.

### Biological processes commonly modulated under severe drought stress

At 20 DASI, the papaya plants reached their lowest performance and clearly presented observable foliar damage (Fig. 1a), so we considered this treatment as severe drought stress. At this time-point, genes involved in water deprivation or salt stress, which are closely related stresses, were enriched in the up-regulated DDEG sets. Additionally, up-regulated DDEGs involved in cold, heat, starvation, and hypoxia stress were enriched, (Supplementary Table S6) indicating a cross-talk between different stress pathways. Furthermore, response to ABA, and oxidation-reduction processes were also up-regulated. On the other hand, several terms related to abiotic stress, ABA (and several hormones), and oxidation-reduction were enriched in the down-regulated gene sets. GO terms related to biotic stress were also down-regulated in all samples. Moreover, plant cell wall composition and elasticity are modulated in response to drought and other stresses^46^. We found “plant cell metabolism” among the enriched GO terms in the down-regulated gene set, which may indicate that roots are adapting to drought stress.

### Long-distance movement of mRNA under drought conditions

The study of the long-distant movement of mRNA through vascular system, most precisely in sap phloem, has attracted more attention in recent years focusing mainly on plant development and response to pathogens attack^15,47,48^. However, little attention has been paid under abiotic stress. In this work, papaya sap transcriptome presented a unique profile in comparison to leaves and roots (Supplementary Fig. S5a,c), showing a large number of genes (3,527) that were specifically regulated in this vascular tissue, even in non-stress conditions (Supplementary Fig. S3d). Sap profile was enriched in GO terms involved in RNA and protein trafficking, mRNA splicing, mature ribosome assembly, translation initiation, post-translational modifications, and several proteins that resemble a nuclear environment (Fig. 4). This enrichment results are in agreement with previous work by Figueroa and cols^49^., which demonstrated by *in vitro* translation assay, that protein translation could occur in sap.

PlaMoM database^50^ reports 11,440 experimentally confirmed mobile genes in Arabidopsis, which represent 5,234 loci. Through homology comparison (Blast searches) against these Arabidopsis loci, we identified 4,408 papaya gene models (from the total 27,769) that could act as putative mobile genes (mRNAs) (Supplementary Table S2). According to ClueGo anaylsis, this set of putative mobile mRNAs were enriched in biological processes related to photosynthesis, response to hormone, response to temperature stimulus, response to inorganic substance, nucleic acid metabolic process, chromosome organization, cellular macromolecule localization, among others (Supplementary Fig. S15a). According to their Molecular Function (MF), 74 and 88 of these mobile papaya mRNAs were clustered in DNA binding and mRNA binding GO terms, respectively (Supplementary Fig. S15b), which is in accordance to previous studies^48^.

From the totals of DDEGs per tissue, including both up- and down-regulated, (3,566, 3,721, and 4,564 in leaf, sap, and root, respectively), we detected 846, 929, and 995 putative mobile DDEGs in leaf, sap, and root tissues, respectively, (Supplementary Fig. S15c). Interestingly, the tissue-specific set of 435 mobile DDEGs in sap were only enriched in GO terms within the BP category related to response to high/low light intensity and photosynthesis processes (Supplementary Fig. S15c). It is worth mentioning that only 24.9% of the total (3,721) of sap DDEGs found in our transcriptome analysis corresponded with those reported as mobile in Arabidopsis PlaMoM database. We also detected a shared core set of mobile mRNAs in response to drought (Supplementary Fig. S15d). This intersection consisting in 182 mobile DDEGs were enriched in GO terms within the BP category related to heat response, secondary metabolic processes, and regulation of signal transduction (Supplementary Fig. S15d,e). As proposed by Thieme and cols.^47^, long-distance mRNA trafficking, could be a rapid alert to distant tissues to achieve a systemic adaptation of an upcoming adverse condition. Mobile elements represent a source of genetic material that could have important implications for the improvement of drought-tolerance in species, as demonstrated by previous studies^49^.

### Gene co-expression networks and transcriptional hubs

The advent of transcriptome profiling experiments have increased the complexity of biological datasets and their interpretation. Unlocking the immense potential of transcriptome data requires the use of new system-level analysis to reveal meaningful relationships between genes and biological processes, as well as the regulatory mechanisms which control specific responses^23^. Currently, the use of biological networks has become a popular and useful approach for depicting the complex organization of biological systems and deciphering the intricate relationships among genes. This network approach has been applied to gene co-expression matrices from transcriptome data to provide early insights into the functional regulation in a spatiotemporal manner for specific phenomena^51^. Some efforts have been made to optimize these network methods and build optimal GCN from transcriptomes data^52,53^. We constructed two independently GCNs for leaves and roots of papaya plant to infer gene responses and relationships during drought, and gain insights into their intricate regulation during stress. The “TF-stress responsive genes” co-expression networks were built on the basis of hub genes^54^, which control abiotic stress responses. A similar strategy was successfully applied on *Xerophyta viscosa* transcriptome to identify key genes in drought response^55^. Through such analysis, the authors found that orthologues of the seed maturation regulators ABI3 and ABI5 played a key role in drought tolerance in vegetative tissues. Furthermore, they suggest that this desiccation tolerance trait in the vegetative tissues of *X. viscosa* comes from a desiccation-tolerance seed character^55^. Our results regarding the GCNs in leaves and roots of papaya plant, demonstrate the potential of this strategy to detect gene modules during drought conditions and to identify gene regulators that play a key role during stress conditions (Supplementary Figs S12 and S13). Besides this, these networks can contain genes of interest for studies about drought responses in tropical plants. We propose a list of 17 candidate TFs that can be used in future work (CRISPR/Cas9 gene edition, knock-out studies, and mutant plant phenotypes analysis) to evaluate their participation in conferring stress tolerance in papaya plant as well as other agro-economically important crops.

## Conclusions

This study provided pivotal insight into the biological processes regulated under moderate and severe drought stress in papaya plants. Tissue-specific genes under CN condition and in response to drought were identified, and the analysis of their up-stream genomic regions could lead to the development of tissue and drought specific promoters. This study also yielded a list of drought responsive genes and TFs as candidates for future functional analyses in papaya. Furthermore, the analysis of papaya plant transcriptomes by GCNs provided a clear concept of the modules, or gene communities, that interact during drought within a specific tissue and at a specific point in time, and suggest the regulatory mechanisms that the papaya plant utilizes to cope with abiotic stress.

## Materials and Methods

### Plant material and experimental design

Seeds of papaya (*Carica papaya* L.) ‘Maradol roja’, acquired from Semillas del Caribe® (Guadalajara, Mexico), were sown in separate substrate-filled pots. After germination, the papaya plants were grown under greenhouse conditions and equivalently watered. Drought stress was imposed on three-month-old plants by withholding watering; healthy well-watered plants of same age were used as controls. Visual assessment of phenotype, physiological measurements, and sample collection for RNA isolation, were performed on plants under control condition (CN), and at 10 and 20 days after stress imposition (DASI).

### Physiological measurements

Physiological measurements were performed on the second fully expanded leaf of three independent papaya plants. Photosynthetic (*A*) and transpiration (*T*) rates were determined by means of a portable Li-6400 photosynthetic system (Li-Cor, Lincoln, NE, USA). Leaf water potential (*Ψ*) was determined by means of a Wescor thermocouple psychrometer sample chamber C-52 connected to a Wescor HR-33T Dew point microvoltmeter (Wescor Inc., Logan, UT, USA).

### RNA isolation and Illumina sequencing

Samples from leaf, sap, and root tissues were collected from two independent plants, immediately frozen in liquid nitrogen, and stored at −80 °C until use. Total RNA from these samples was isolated using TRIzol® reagent (Invitrogen®). Quality and concentration of purified RNA were assessed by 1% agarose gel electrophoresis and a 2100 Bioanalyzer (Agilent Technologies®). The cDNA libraries were sequenced at the Genomic Services Laboratory, Advanced Genomic Unit (UGA,Cinvestav-Langebio), Mexico. A total of 18 cDNA Libraries (Table 1) were prepared using the Illumina® TruSeq® RNA Sample Prep Kit v2, and paired-end sequenced in a 2 × 75 High Output configuration on the NextSeq500 Illumina platform. Quality check of RNA-seq raw reads was performed by means of FastQC^56^ software. Trimmomatic v0.36^57^ was used for adapter clipping and filtering, and only reads with a score >Q30 and a minimum length of 54 pb were kept (clean reads) for further analyses.

### *De novo* transcriptome assembly and annotation

The clean reads of the 18 sequenced cDNA libraries were used to construct a *de novo* transcriptome assembly by means of Trinity v2.2.0^36^ with default options. The longest isoform of each assembled unigene was annotated with Blast2GO^58^. Annotations were based on BLASTX^59^ similarity searches against the Plant ref-seq protein database of the NCBI (ftp://ftp.ncbi.nlm.nih.gov/refseq/release/plant/*protein.faa.gz). BLASTX search parameters were: HSP cut-off length 33, report 20 hits, maximum E-value 1e-3. Blast2GO Mapping and annotation parameters were: E-value 1e-6, annotation cut-off 55, GO weight 5, HSP-hit coverage cut-off 20. Additionally, the unigenes were blasted against the gene models of the reference genome of the transgenic ‘SunUp’ papaya^37^, to find the correspondence between them. Furthermore, putative coding regions (>100 bp) from the unigenes were detected by means of Transdecoder v3.0.0 (https://github.com/TransDecoder/TransDecoder/wiki) with default options.

### Expression level quantification

Expression level quantification was performed using both *de novo* and reference assembly approaches. For the *de novo* transcriptome analysis, expression quantification was estimated by mapping the 18 cDNA libraries (clean reads) to the assembled transcriptome by means of Bowtie2^60^ and RNA-Seq by Expectation-Maximization (RSEM) software v1.2.27^61^, using the scripts included in the Trinity package^62^. For the reference-based transcriptome analysis, the 18 cDNA libraries (clean reads) were mapped to the reference genome of the transgenic ‘SunUp’ papaya^37^, downloaded from Phytozome v12.1^63^, by means of TopHat^64^ and HTSeq^65^. Read counts resulting from the *de novo* (unigenes) and reference-based (genes) transcriptomes were normalized to transcripts per million (TPM).

### Differential expression analysis

Two series of differential expression analyses were performed for both the reference and *de novo* assembly approaches. Firstly, leaves, sap, and roots samples were compared under control condition, i.e L-CN = L-CN_vs_S-CN and L-CN_vs_R-CN; S-CN = S-CN_vs_L-CN and S-CN_vs_R-CN; R-CN = R-CN_vs_L-CN and R-CN_vs_s-CN (Fig. 2 and Supplementary Figures S3 and S6). Secondly, control samples were compared against the stressed samples, i.e L-10 = L-CN_vs_L-10; L-20 = L-CN_vs_L-20; S-10 = S-CN_vs_S-10; S-20 = S-CN_vs_S-20; R-10 = R-CN_vs_R-10; R-20 = R-CN_vs_R-20 (Fig. 2 and Supplementary Figs 4 and 7. Only read counts of genes and unigenes with TPM values of ≥1 and ≥16 (parameters established based on linear regression analysis), respectively, were used for differential expression analysis by means of EdgeR^66^ in R v3.0^67^. Differentially expressed genes (DEGs) or unigenes (DEUs) were defined as those presenting an absolute fold change (FC) ≥2 and an adjusted P-value (FDR) ≤ 0.001 in any pairwise comparison.

### GO functional enrichment analysis of sets of differentially expressed genes

For each set of DEGs obtained from the reference-based transcriptomic analysis, significant enriched GO terms (in the Biological Process category), based on Plaza 4.0^68^ annotations, were detected by means of the Fisher’s exact test (FDR < 0.05) implemented in Blast2GO^58^. Specific sets of DEGs under control condition (Supplementary Fig. S3d) were compared against each other. Sets of up- and down-regulated DDEGs, identified in the reference-based transcriptomic analysis, were separately submitted to GO enrichment analysis by comparing them to the total expressed genes (TPM ≥ 1) in leaf, sap, and roots under CN condition. Then, enriched GO terms and genes (Arabidopsis identifiers) were clustered and visualized as networks by means of ClueGO v2.5.0^69^ in Cytoscape v3.6.0^70^.

### Gene co-expression network analysis

TPM values of the sets of up-regulated genes (obtained from the reference-based transcriptomic analysis) found in leaves and roots (but not sap) at 10 and 20 DASI were used to build two tissue-specific pairwise co-expression matrices by means of the GENIE3 Bioconductor package^71,72^, which is based in Random Forest machine learning model^73^, using default parameters. A total of 95 papaya gene models corresponding to transcription factors (TFs), 39 in leaves (Supplementary Table S7) and 56 in roots (Supplementary Table S8), from the principal families implied in responses to abiotic stress (AP2/ERF, AREB/ABF, NAC, MYB, bHLH, WRKY, and HSF [Heat shock factor]) were set as “Regulator nodes”, and all of the up-regulated DDEGs (leaves and roots) were set as “Target nodes” for the building of these “TF-stress responsive genes” networks. Identified expression patterns were taken as an indication of putative regulatory links. All connections (links) between genes (nodes) were exported as tables and loaded into Cytoscape v.3.6.0^70^, and they can be found in Supplementary Tables S9 and S10. Calculation of network indices and parameters were performed using the NetworkAnalyzer built-in app in Cytoscape and can be inspected in the Supplementary Tables S7 and S8. The networks were taken as undirected but weighted. Network clusters or communities were determined based on topological edge connections using the GLay^74^ network clustering algorithm plug-in in Cytoscape. Final networks were displayed using the yGraph Organic layout and the “Degree” parameter was used to depict the size of the nodes.

### Statistical analysis and data visualization

All data analyses, graphics, and heatmaps were made in R 3.4.4^67^. Significant statistical differences of physiological parameters among treatments were determined with one-way ANOVA test followed by Tukey’s test (P < 0.001). Graphics were made with ggplot2^75^. Venn diagrams were plotted with Vennerable package (https://github.com/js229/Vennerable). Heatmaps were drawn with either Heatmap3^76^ or ComplexHeatmap^77^ packages.

## Electronic supplementary material

Supplementary Figures

Supplementary Figures Legends

Supplemenetary Tables

## Data Availability

RNA-seq raw reads data of the 18 sequenced libraries were deposited in the National Center of Biotechnology Information (NCBI) Sequence Read Archive (SRA accession: SRP145336), under the Bioproject PRJNA470602. The samples are described by Biosamples SAMN09096772-SAMN09096789.
