# Supplementary material for: Transcriptomics and co-expression networks reveal tissue-specific responses and regulatory hubs under mild and severe drought in papaya (Carica papaya L.)
Source: Sci Rep. 2018 Sep 28;8:14539. doi: 10.1038/s41598-018-32904-2 (PMC6162326; doi:10.1038/s41598-018-32904-2)

Title

Transcriptomics and co-expression networks reveal tissue-specific responses and regulatory hubs under mild and severe drought in papaya (*Carica papaya* L.)

Authors

Gamboa-Tuz Samuel David[a,1], Pereira-Santana Alejandro[a,1], Zamora-Briseño Jesús Alejandro[a], Castano Enrique[b], Espadas-Gil Francisco[a], Ayala-Sumuano Jorge Tonatiuh[c,d], Keb-Llanes Miguel Ángel[a], Sanchez-Teyer Felipe[a], and Rodríguez-Zapata Luis Carlos[a,*].

[a] Biotechnology Unit, Yucatan Center for Scientific Research (CICY), 97205, Merida, Yucatan, Mexico.

[b] Plant Biochemistry and Molecular Biology Unit, Yucatan Center for Scientific Research (CICY), 97205, Merida, Yucatan, Mexico.

[c] IDIX S.A. de C.V. Av. Sonterra 3035 int. 26, Querétaro, México

[d] Polytechnic University of Huatusco, 94100, Veracruz, México.

[1] Contributed equally.

* Corresponding author. E-mail: lcrz@cicy.mx.

Supplementary Figure S1

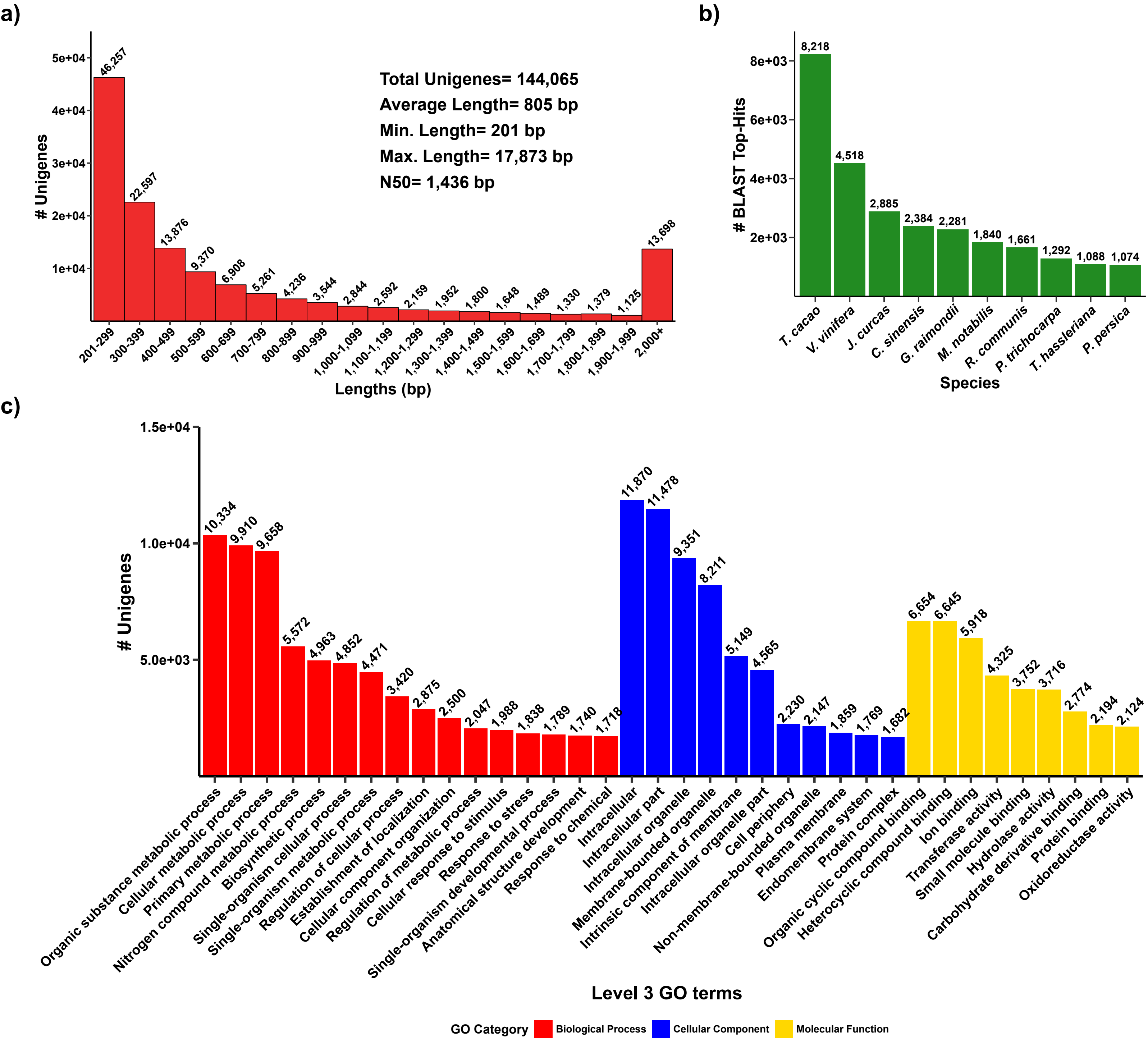

Supplementary Figure S2

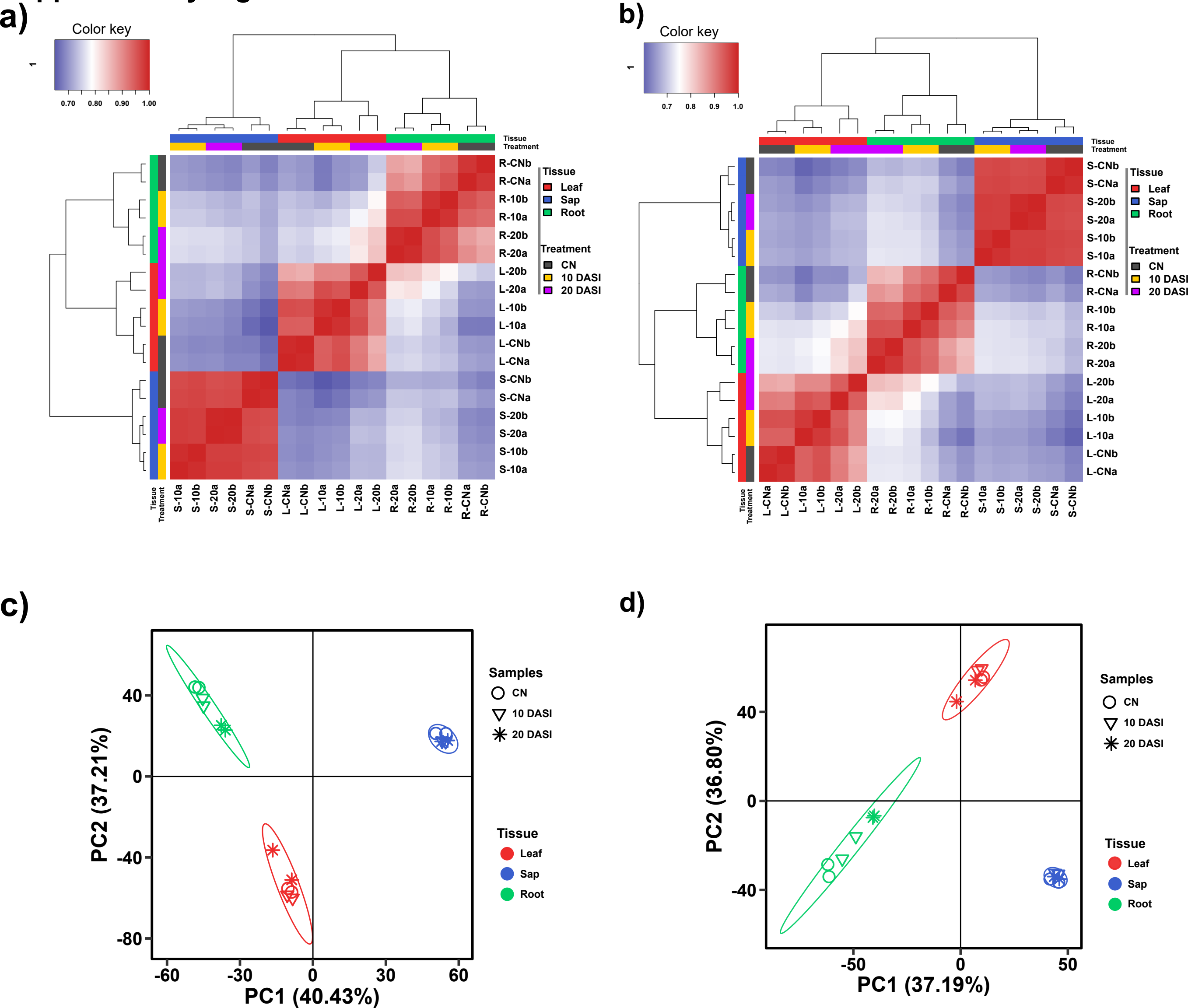

Supplementary Figure S3

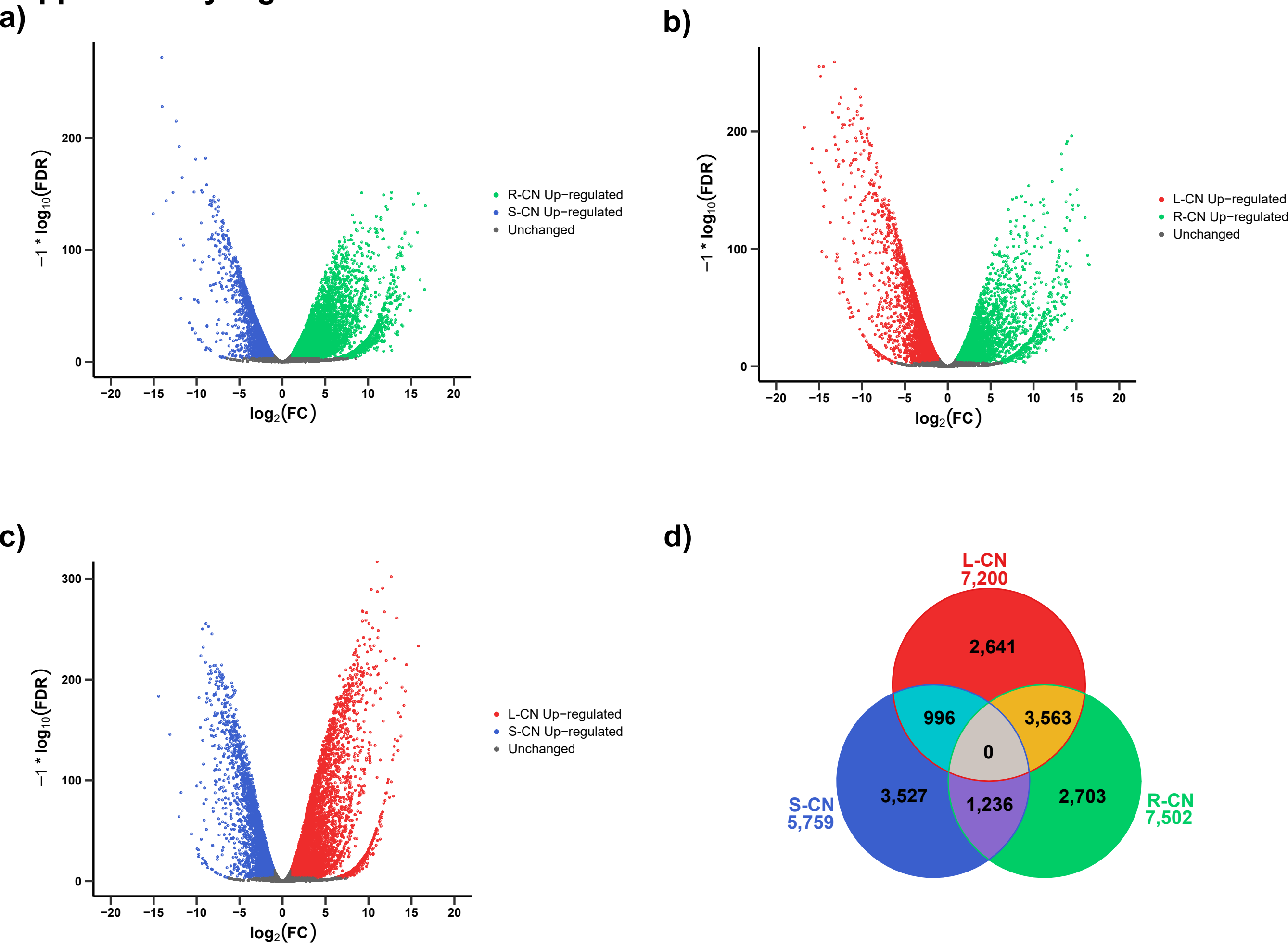

Supplementary Figure S4

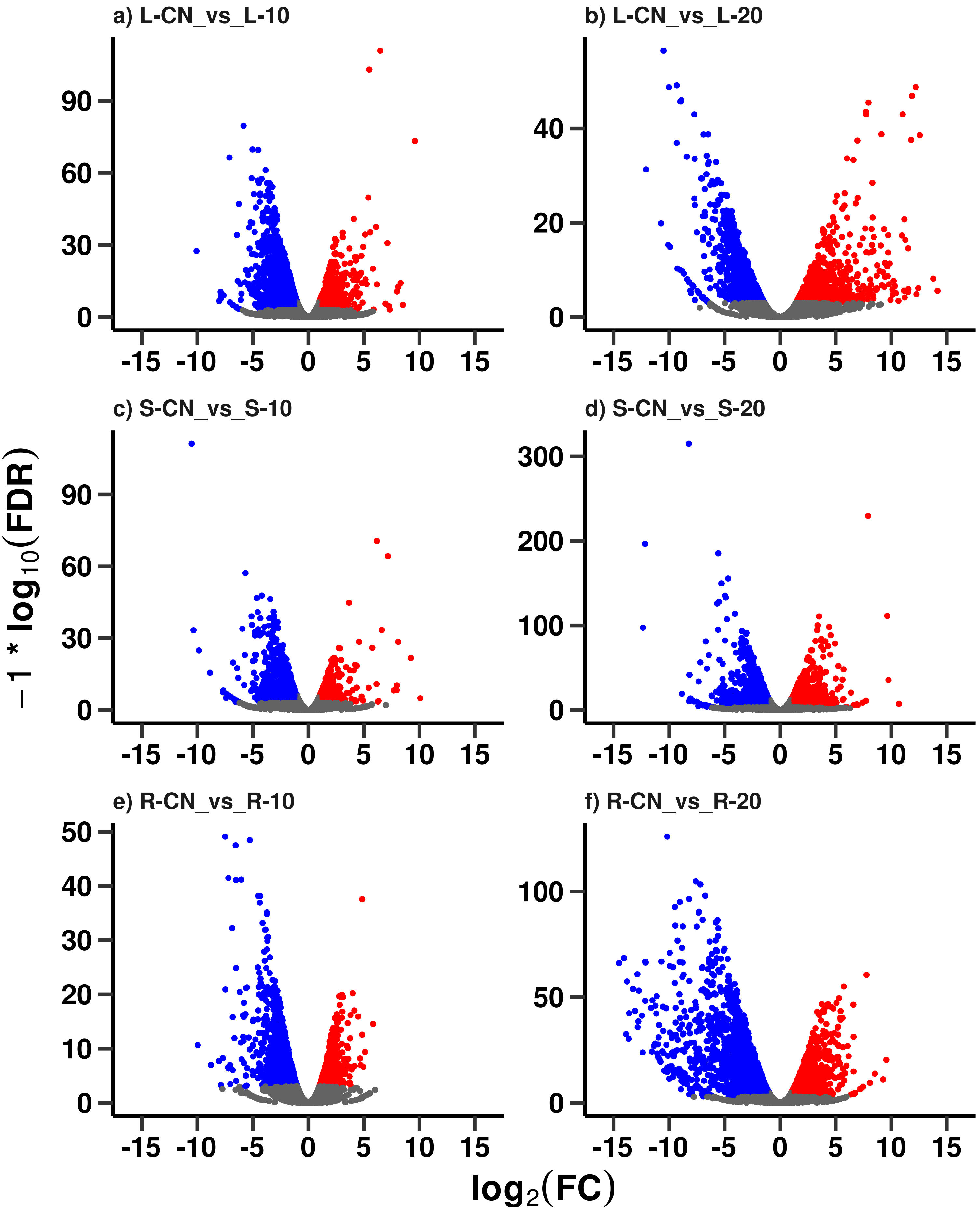

Supplementary Figure S5

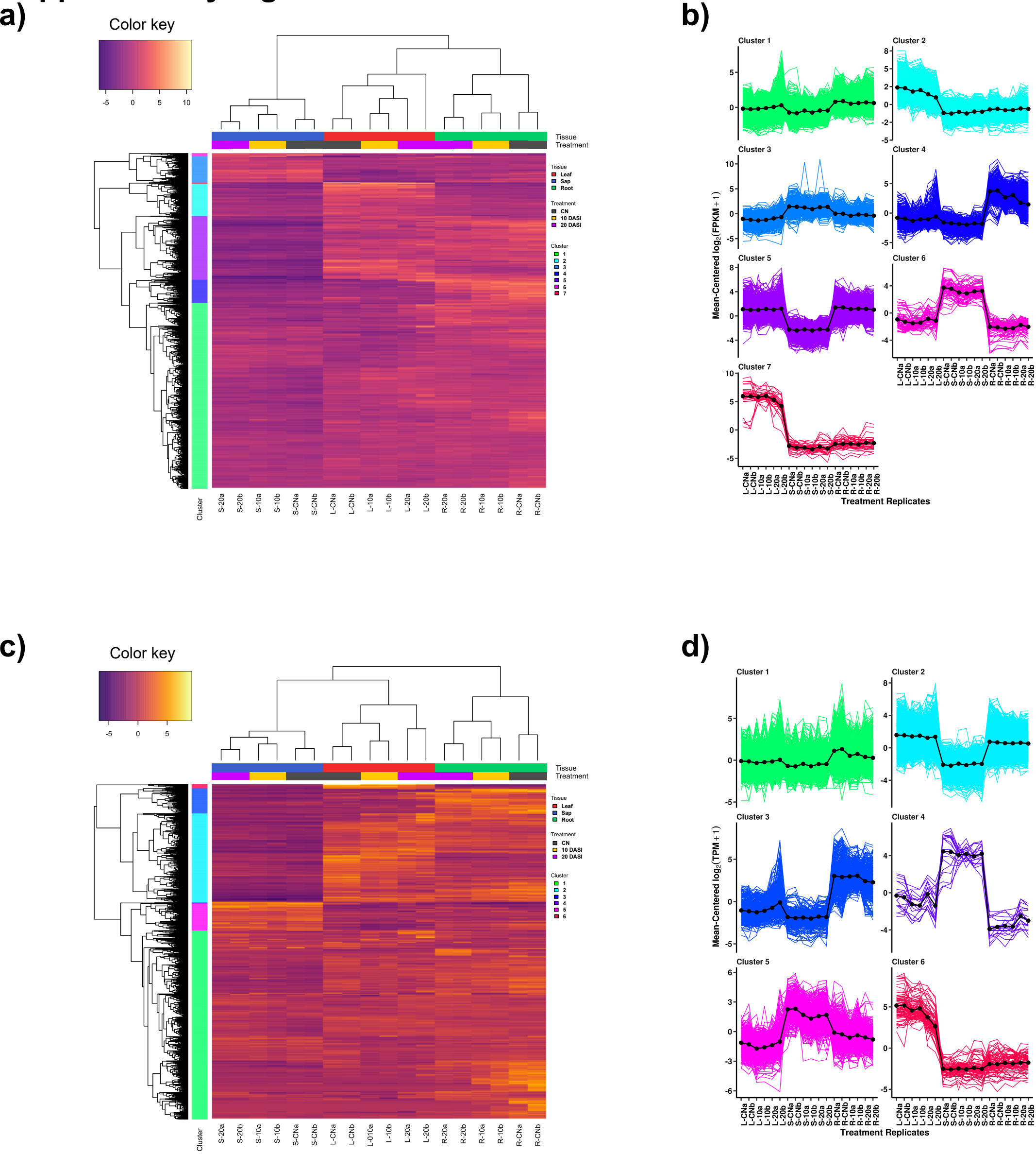

Supplementary Figure S6

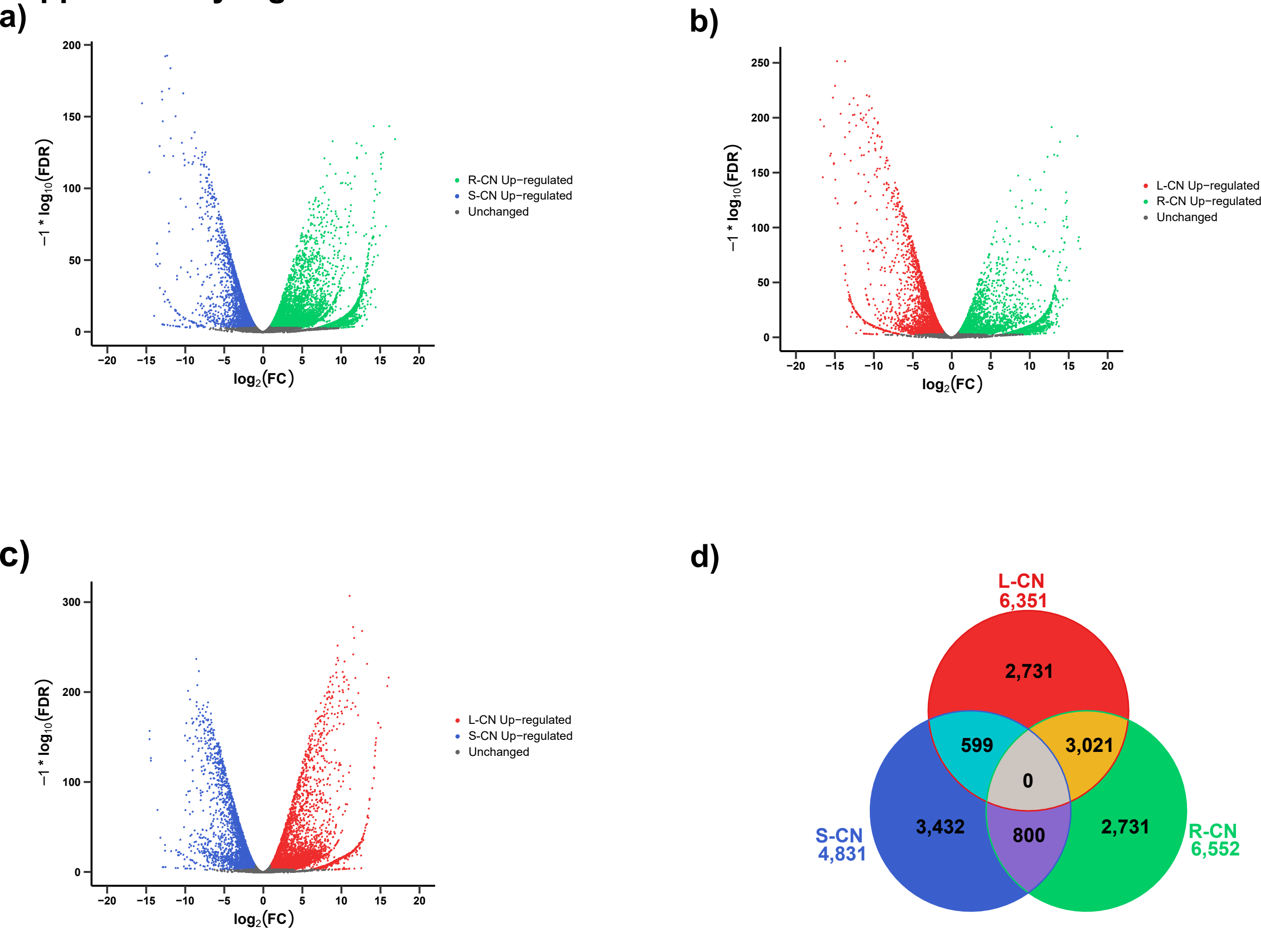

Supplementary Figure S7

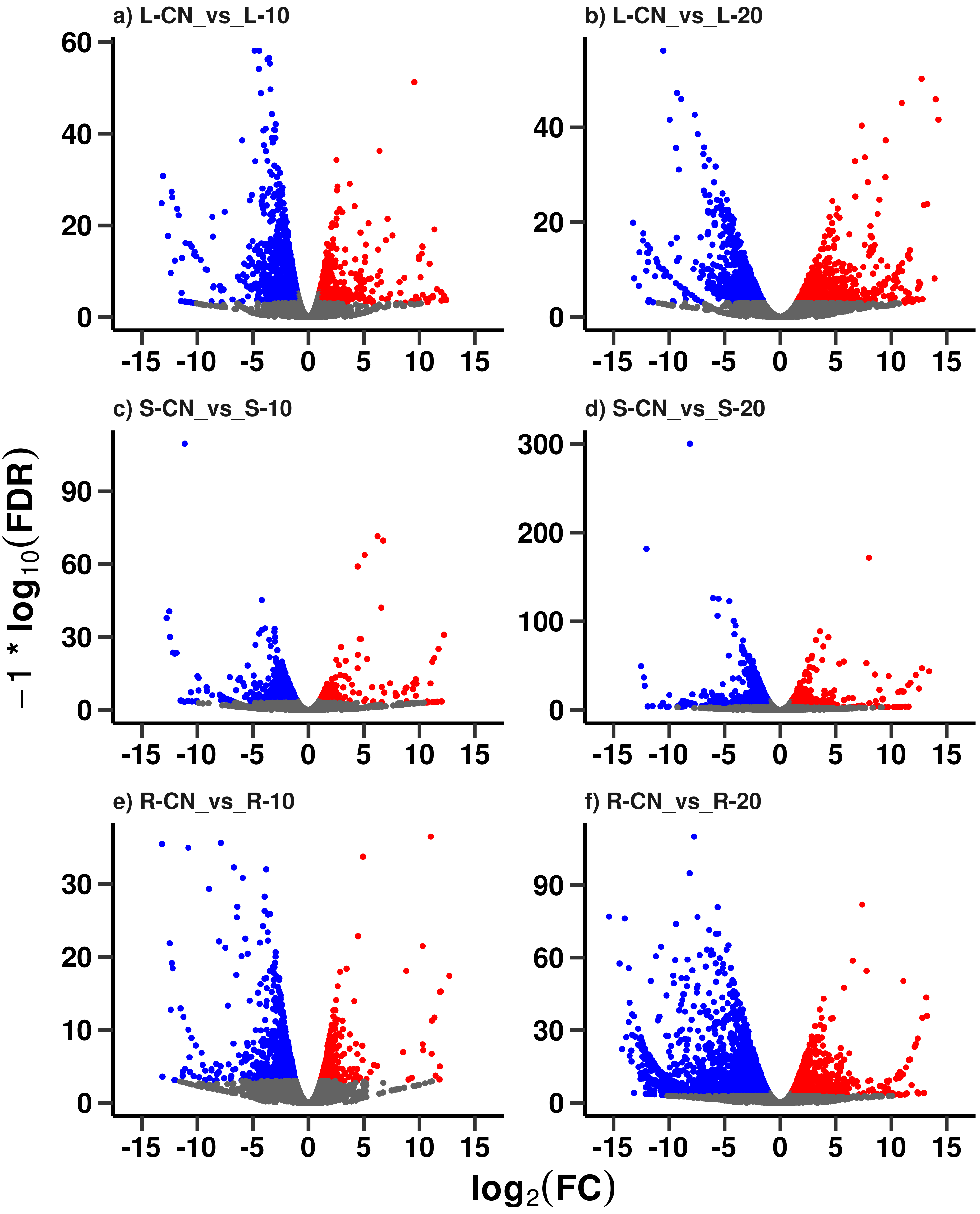

Supplementary Figure S8

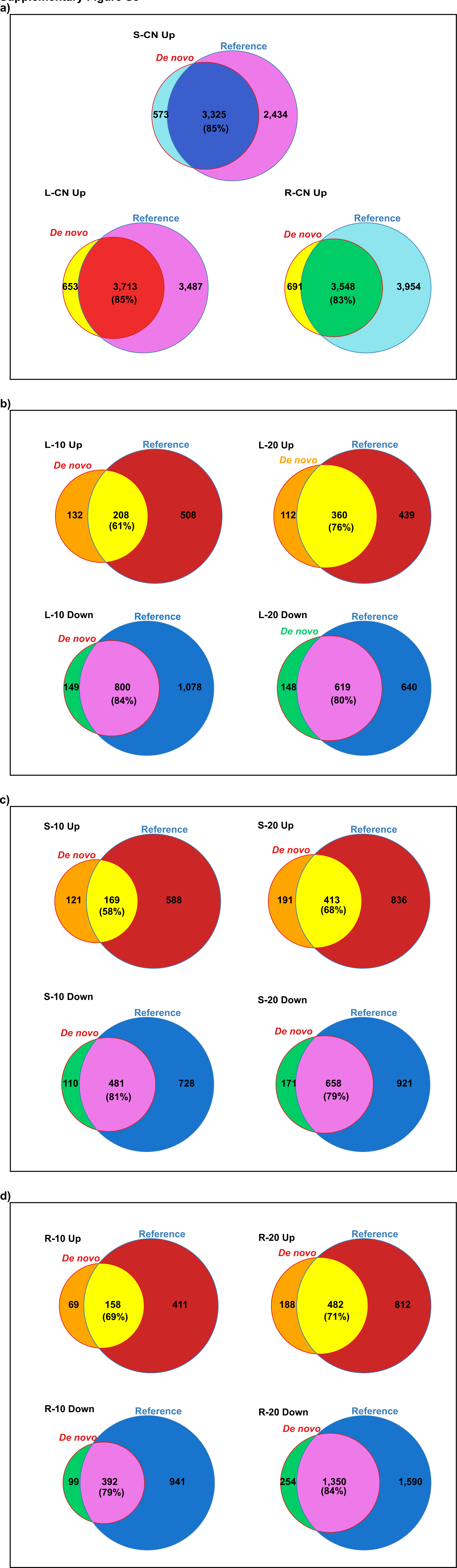

Supplementary Figure S9

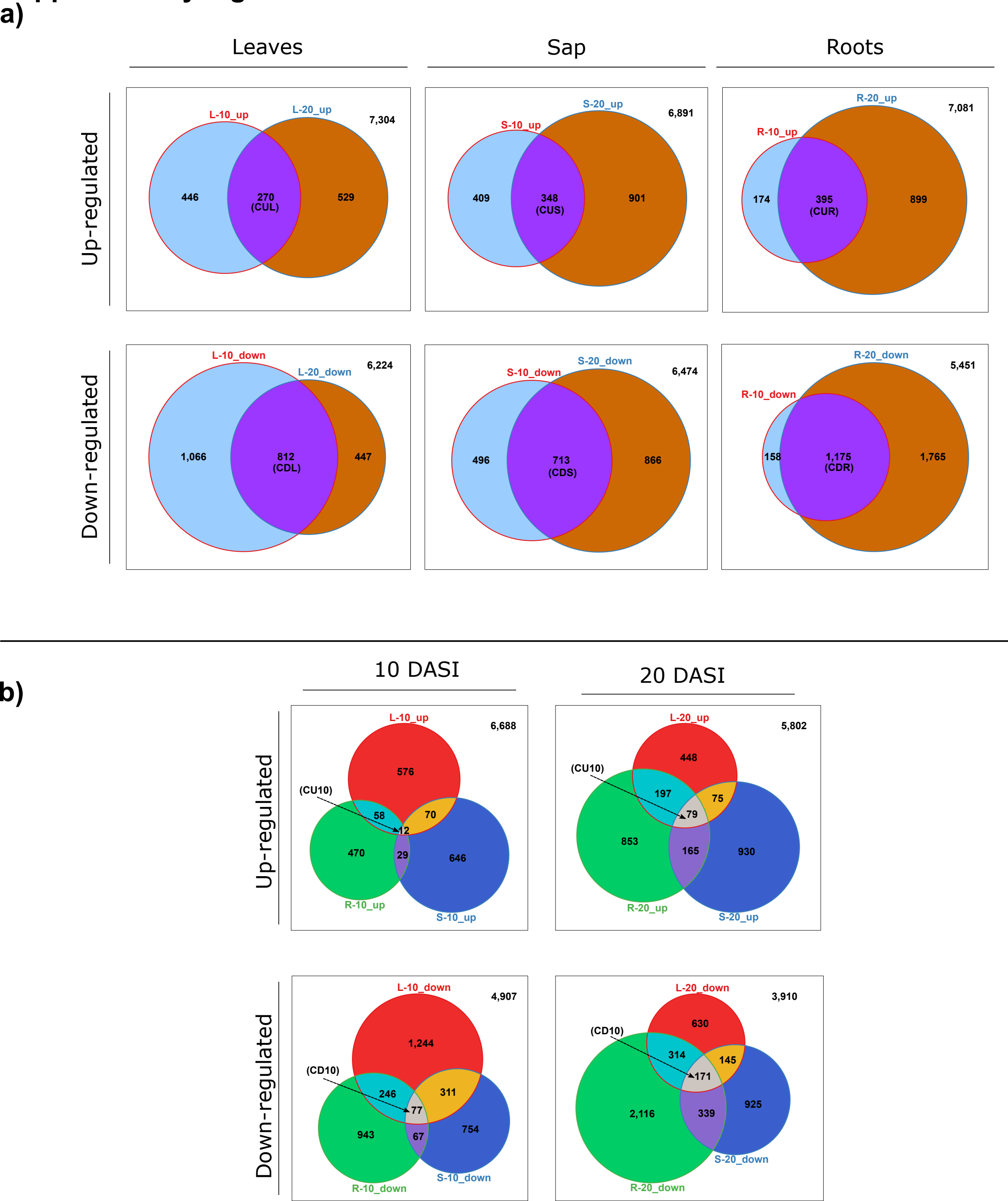

Supplementary Figure S10

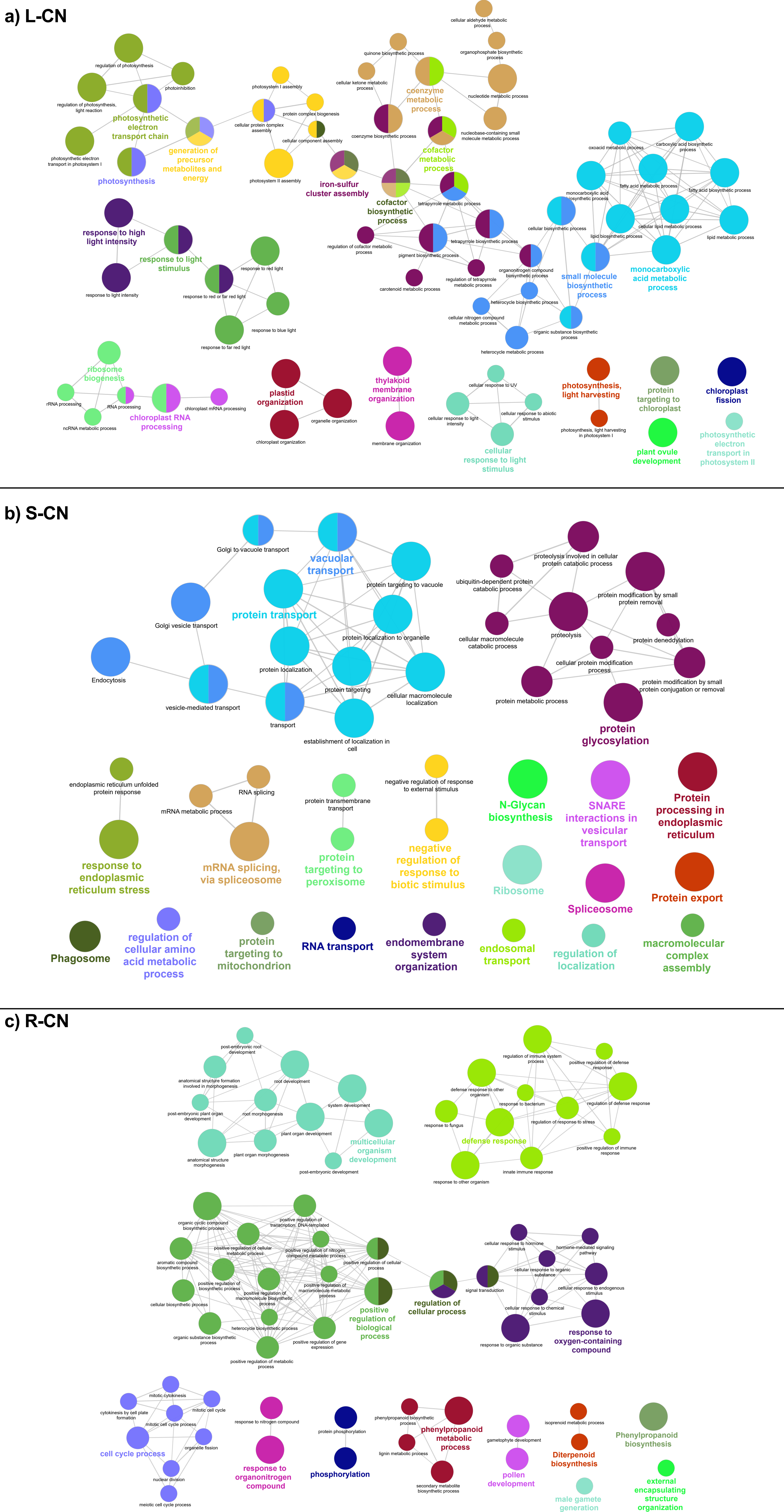

Supplementary Figure S11

a) L-10 Up

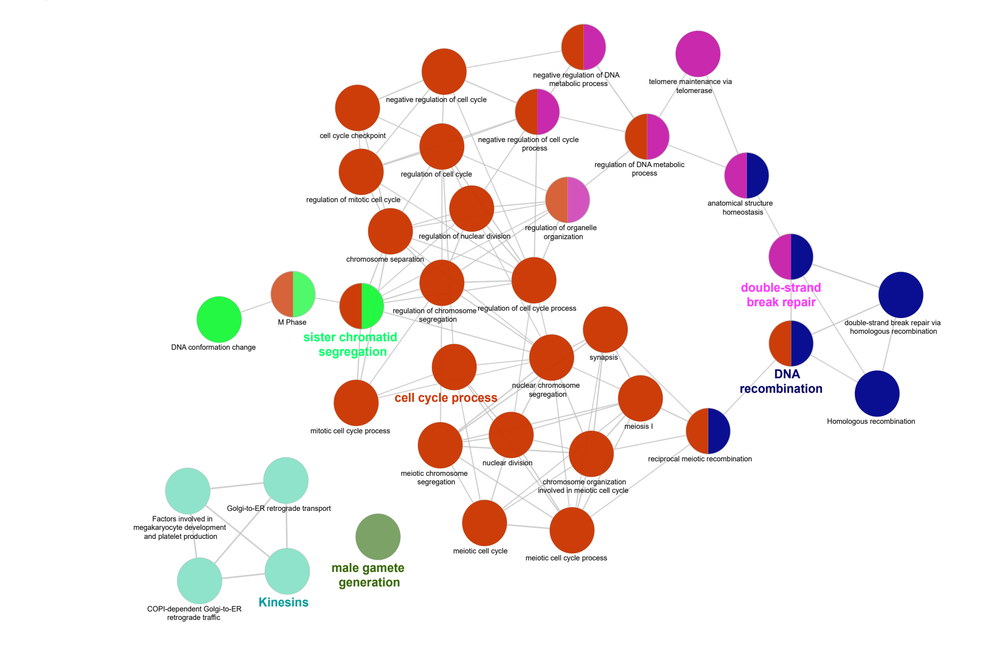

b) S-10 Up

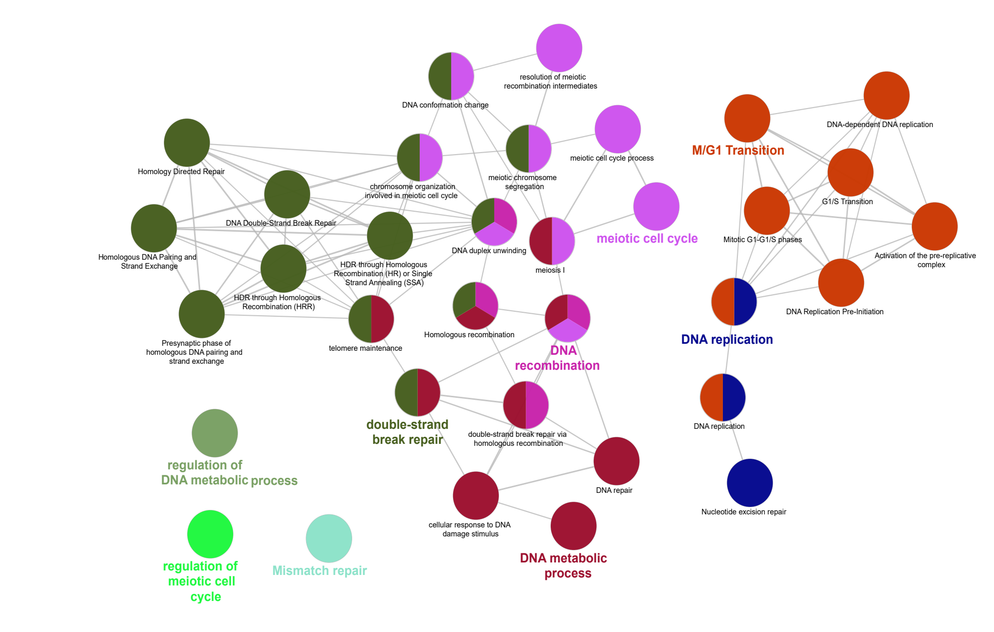

c) R-10 Up

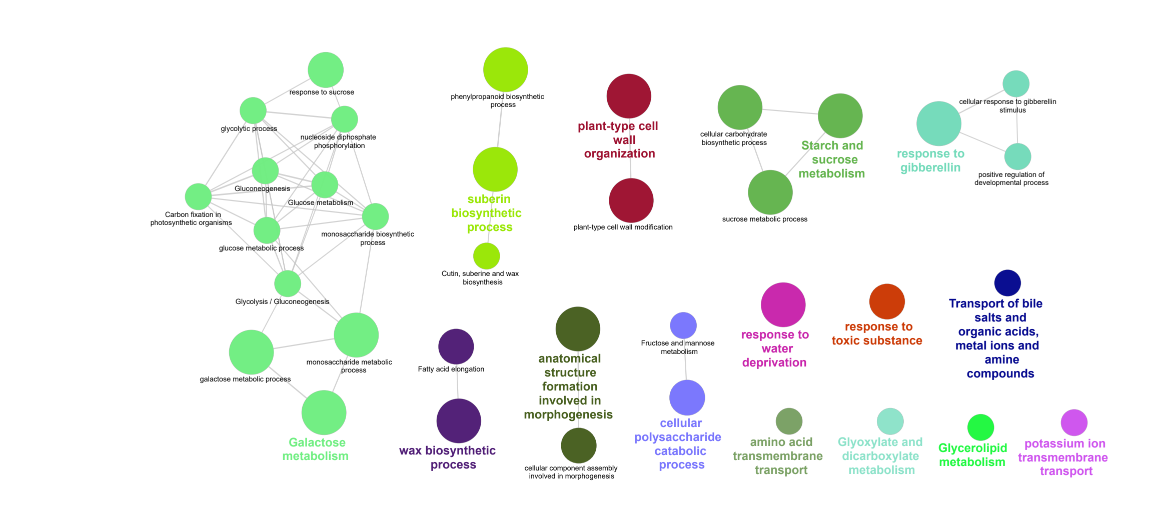

d) L-20 Up

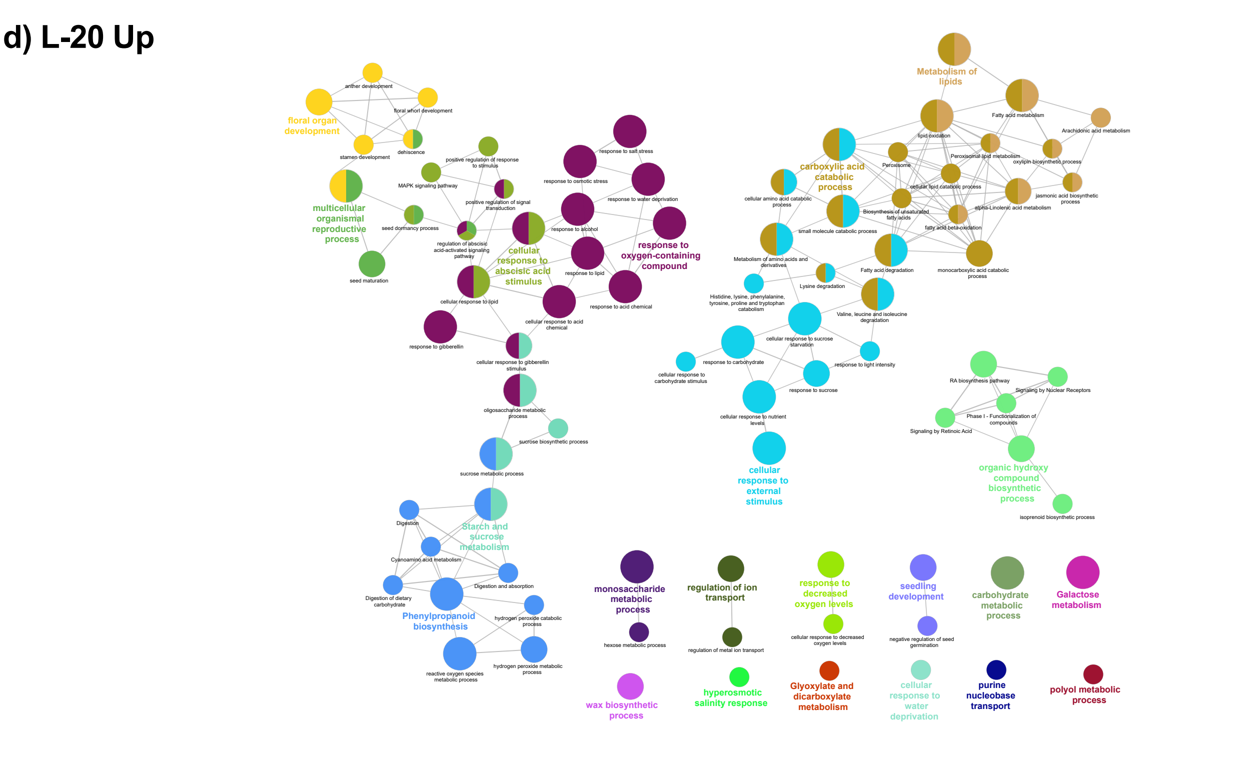

e) S-20 Up

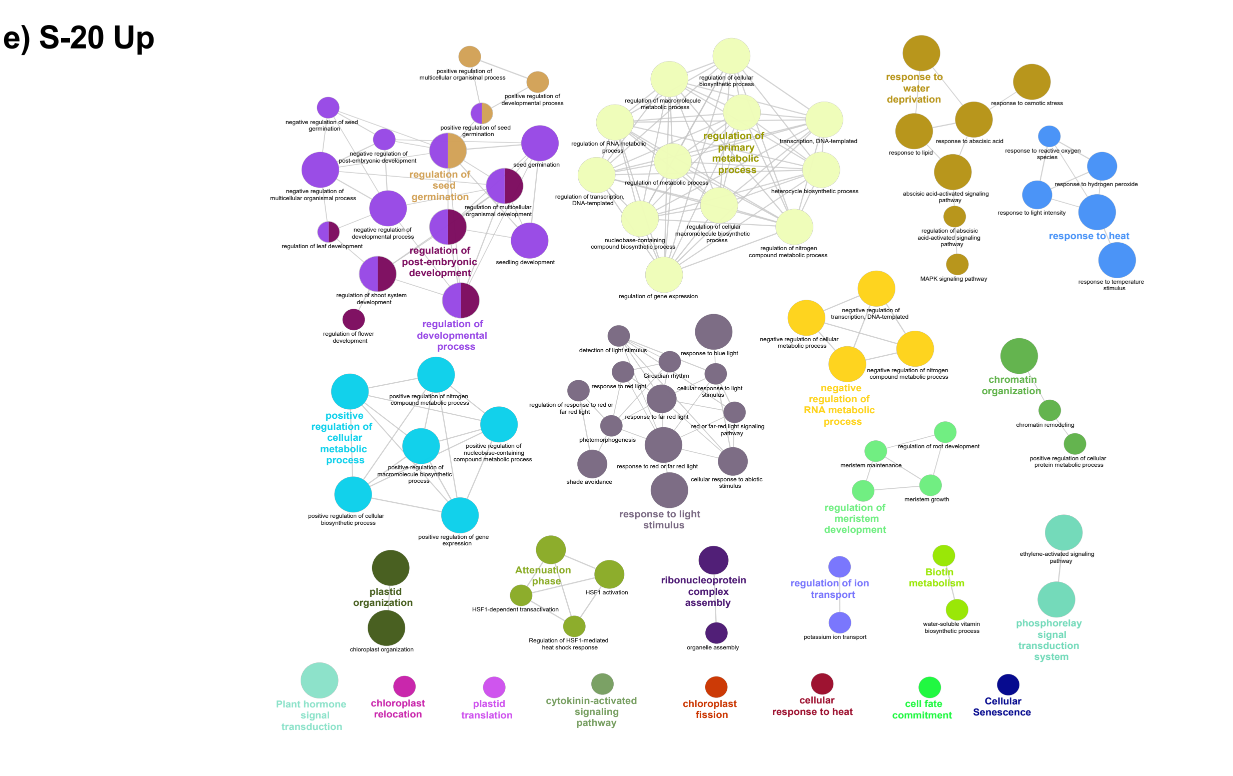

f) R-20 Up

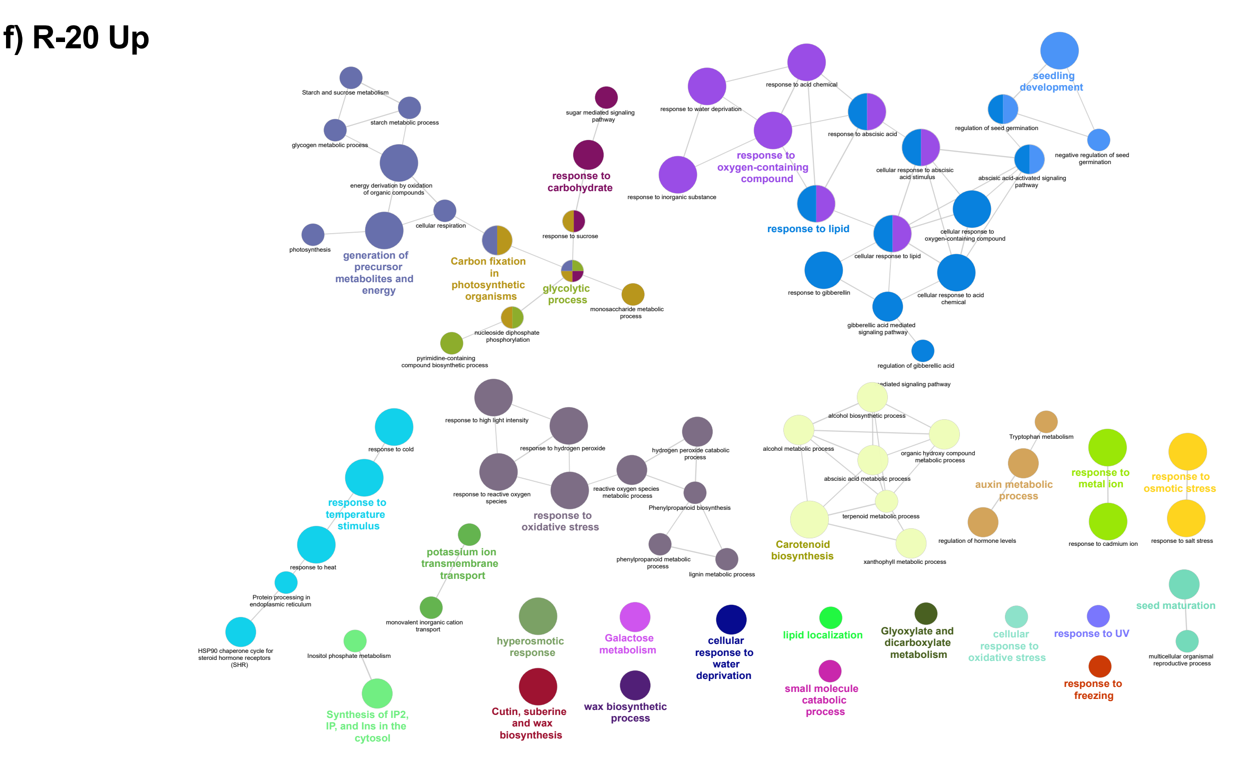

g) L-10 Down

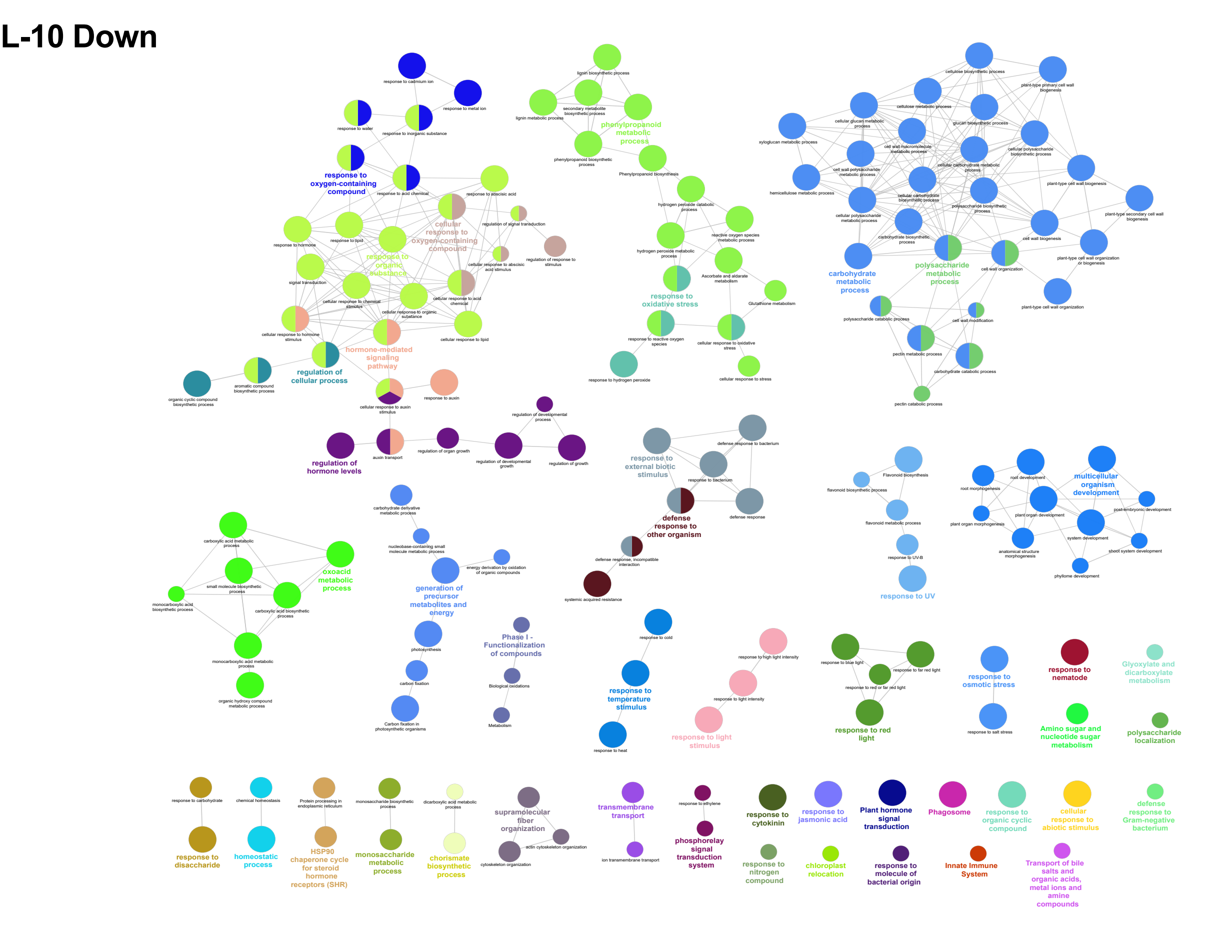

h) S-10 Down

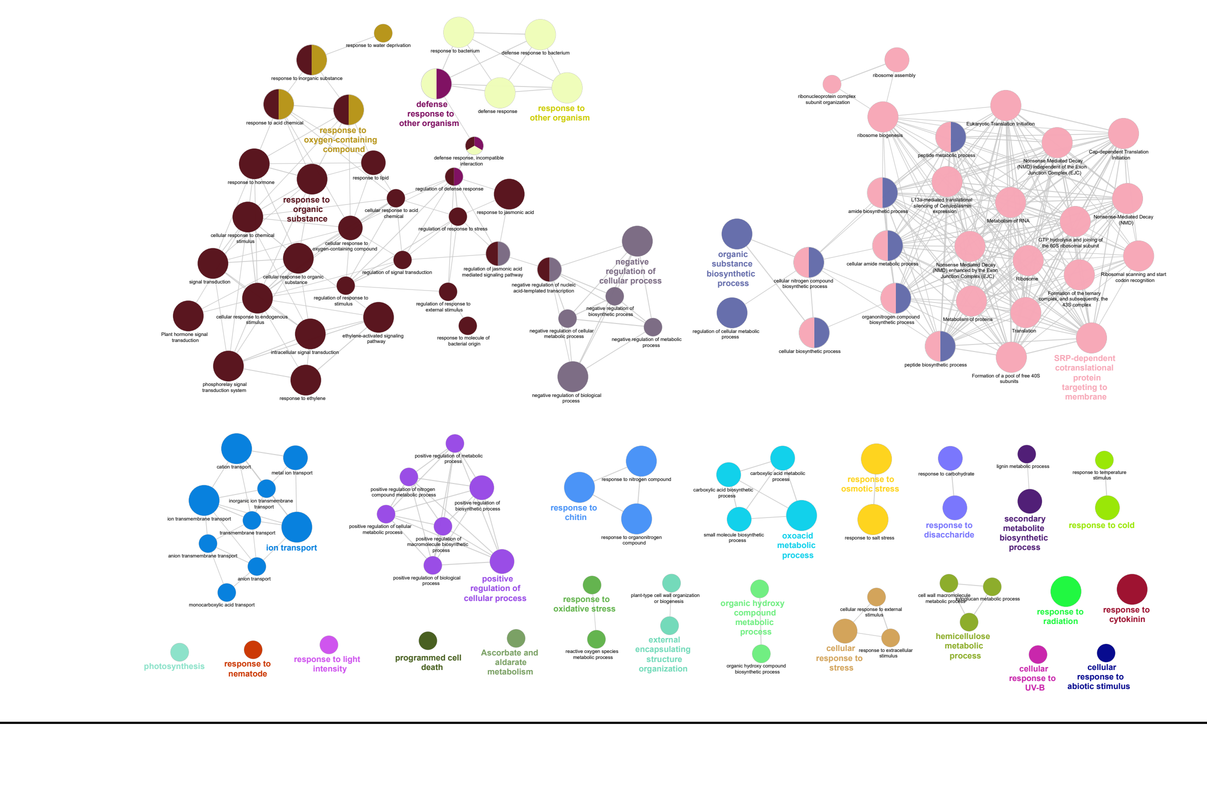

i) R-10 Down

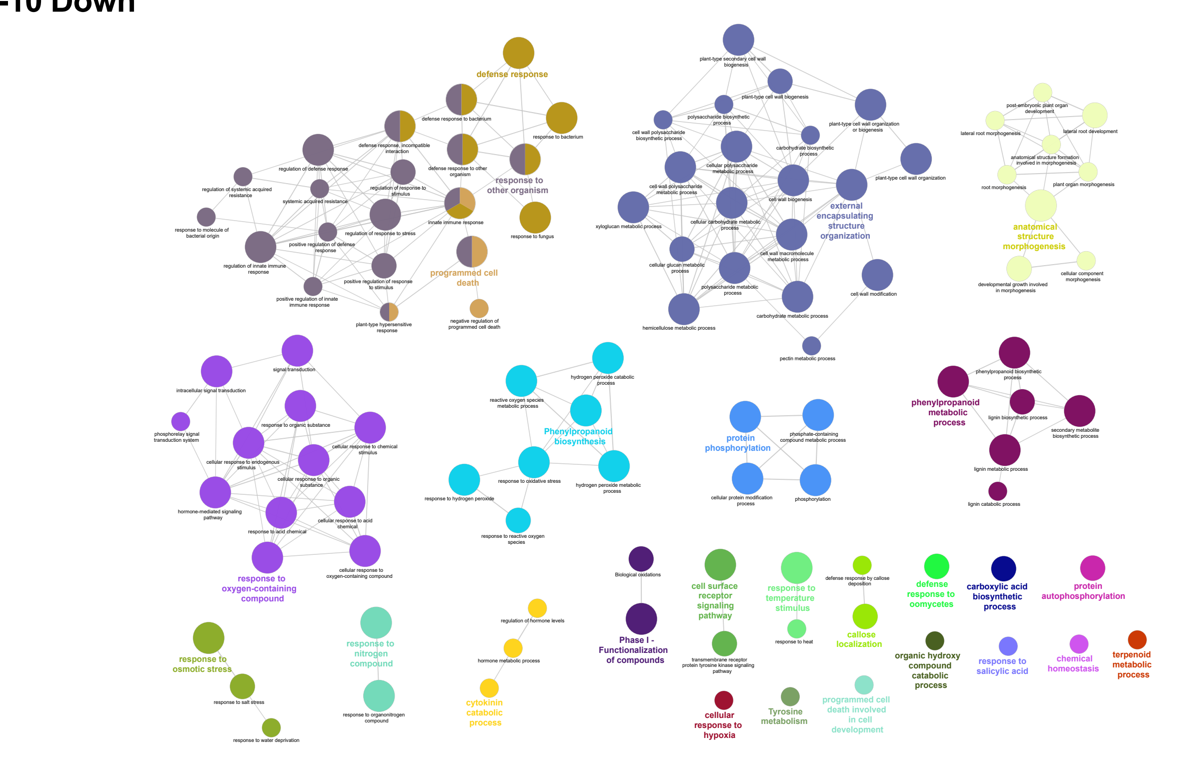

j) L-20 Down

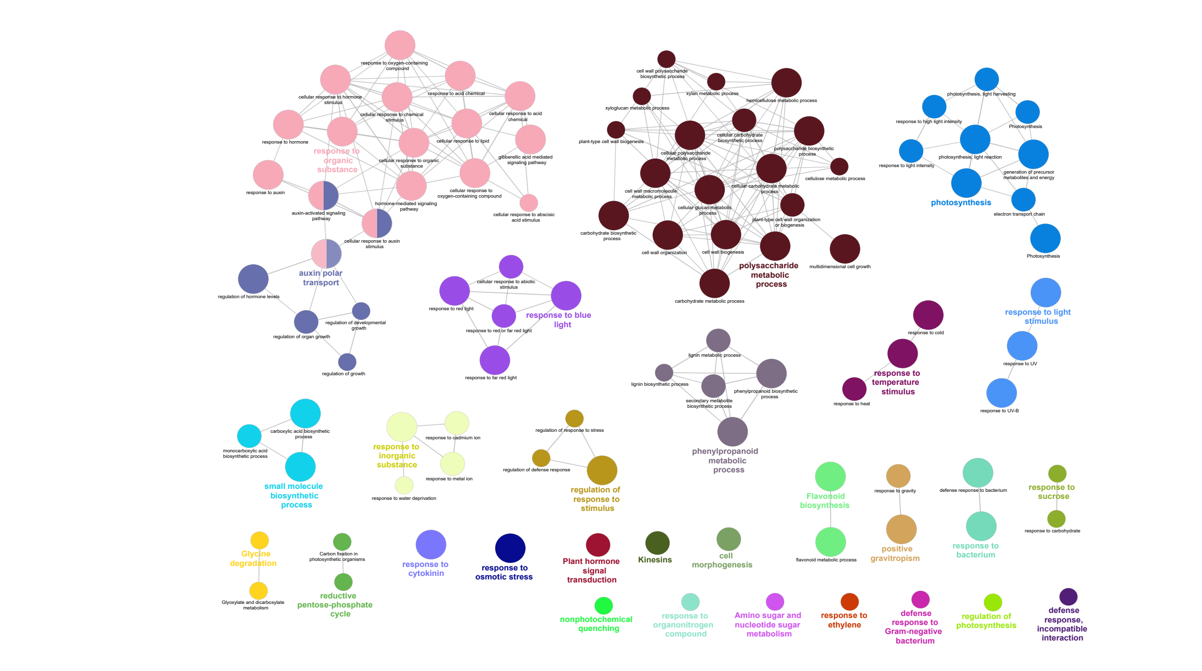

k) S-20 Down

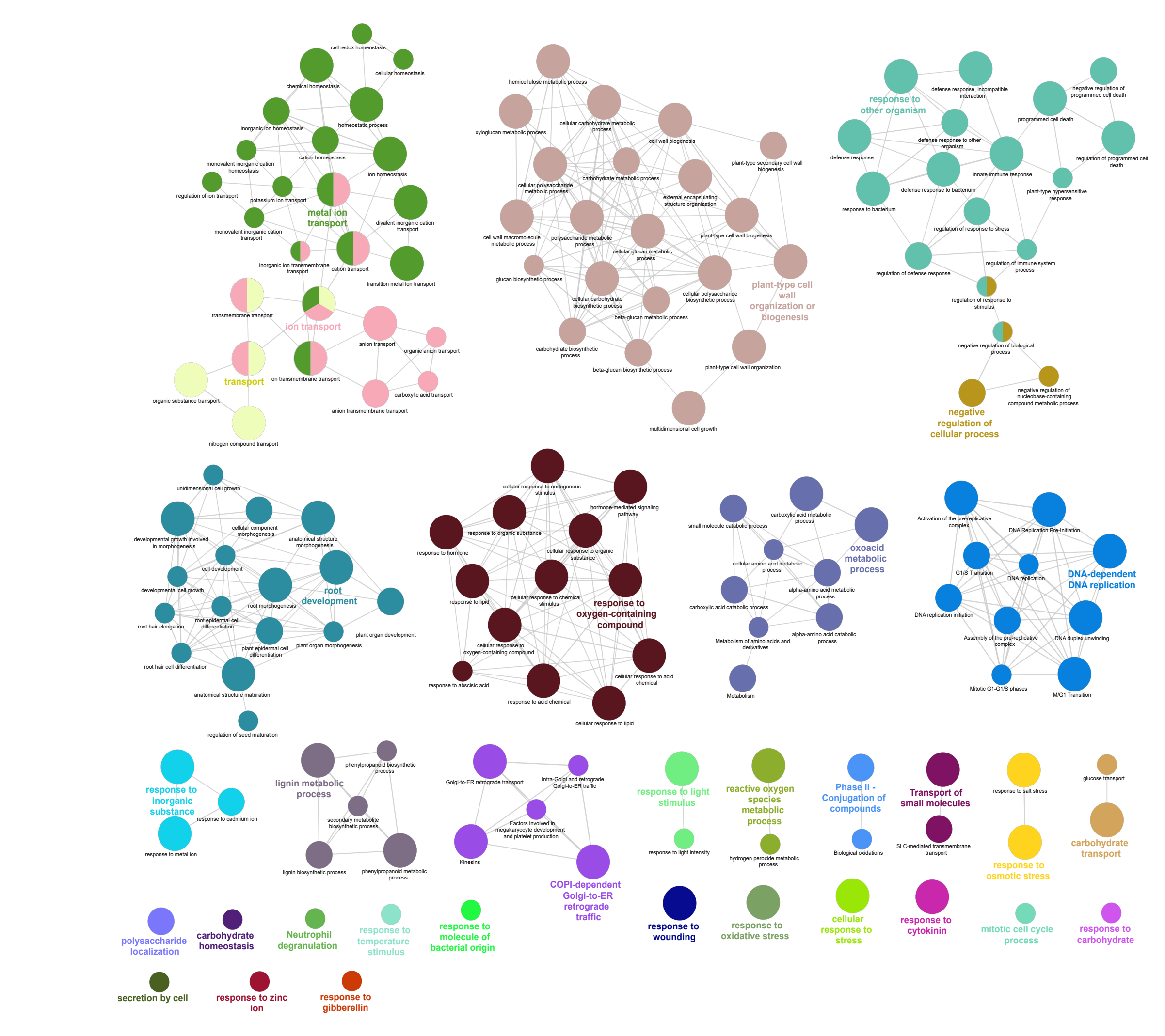

l) R-20 Down

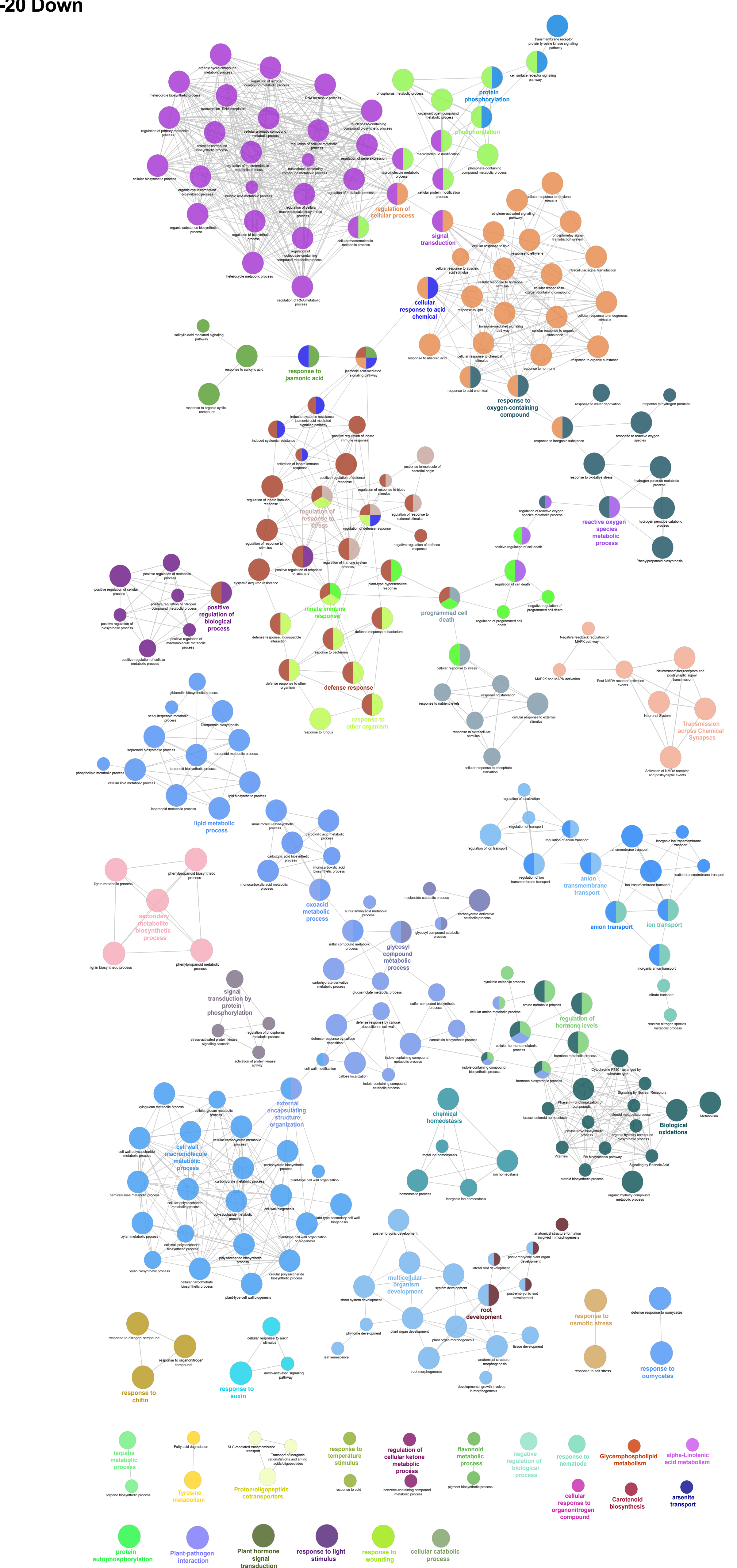

Supplementary Figure S12

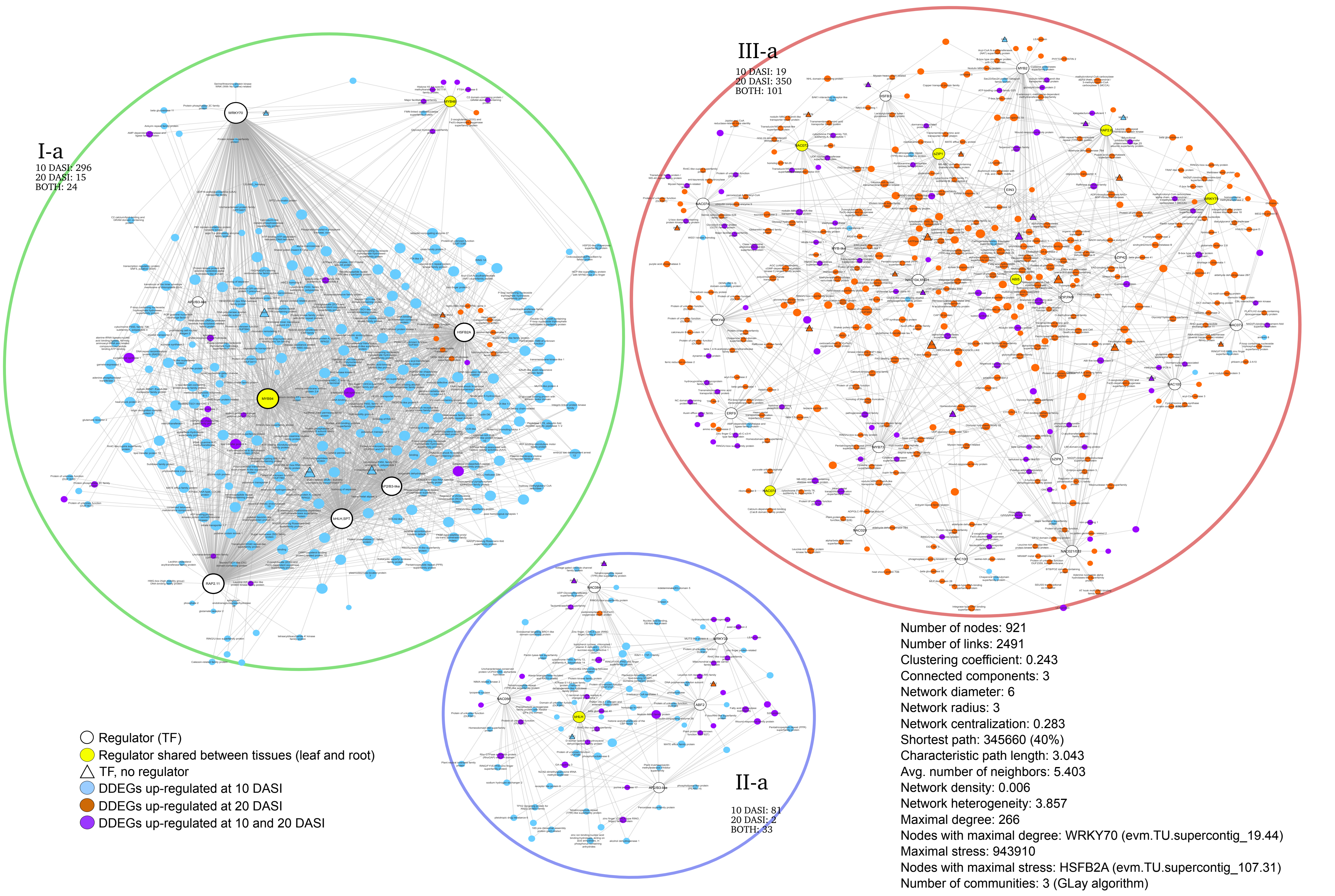

Supplementary Figure S13

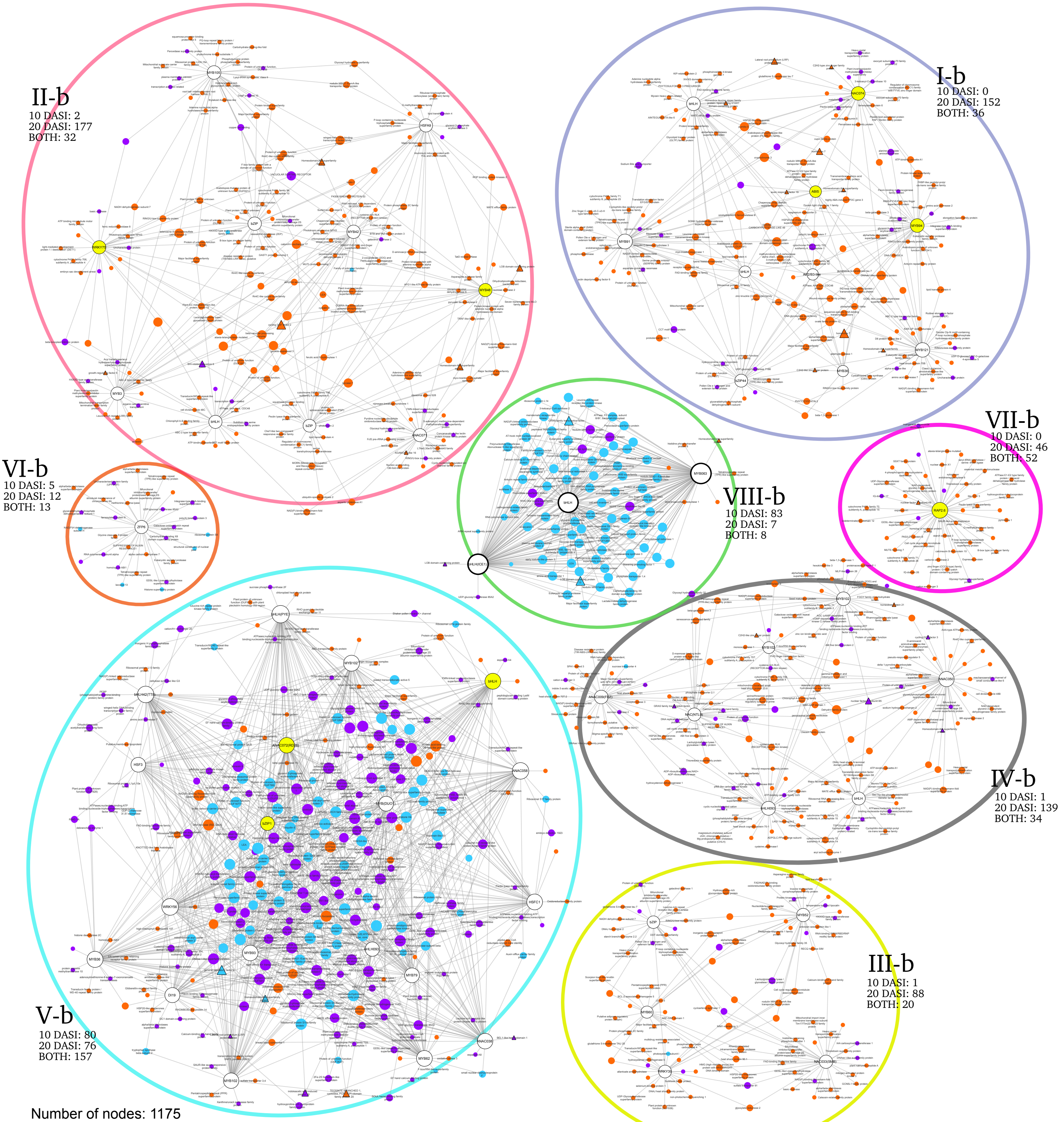

Number of nodes: 1175
Number of links: 2054
Clustering coefficient: 0.106
Connected components: 8
Network diameter: 6
Network radius: 1
Network centralization: 0.075
Shortest path: 232024 (16%)
Characteristic path length: 3.100
Avg. number of neighbors: 3.493
Network density: 0.003
Network heterogeneity: 2.422
Maximal degree: 91
Nodes with maximal degree: MYB63 (evm.TU.supercontig_34.3)
Maximal stress: 169468
Nodes with maximal stress: bHLH42(TT8; evm.TU.supercontig_52.26)
Number of communities: 8 (GLay algorithm)

- Regulator (TF)
- Regulator shared between tissues (leaf and root)
- TF, no regulator
- DDEGs up-regulated at 10 DASI
- DDEGs up-regulated at 20 DASI
- DDEGs up-regulated at 10 and 20 DASI

Supplementary Figure S14

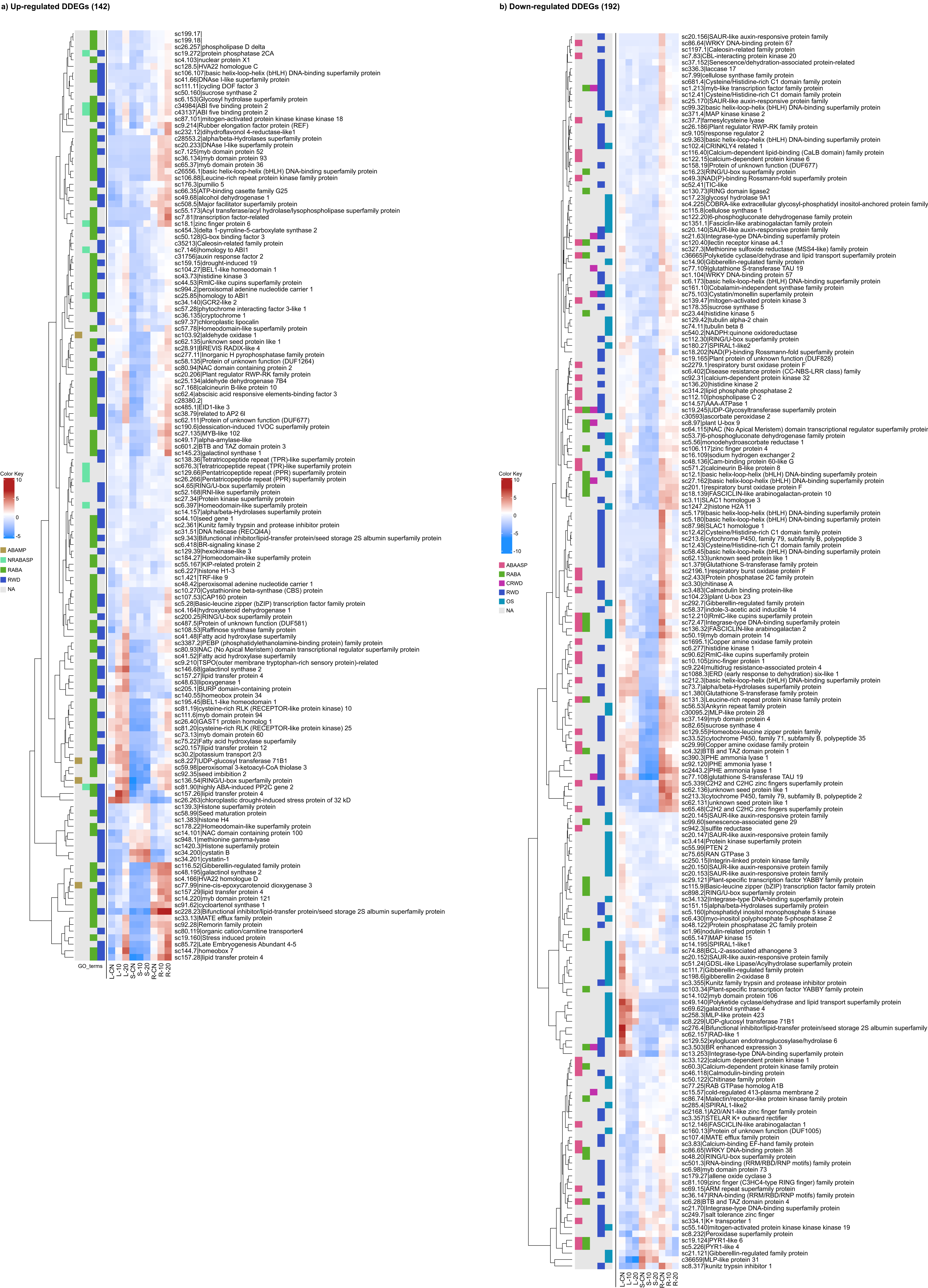

Supplementary Figure S15

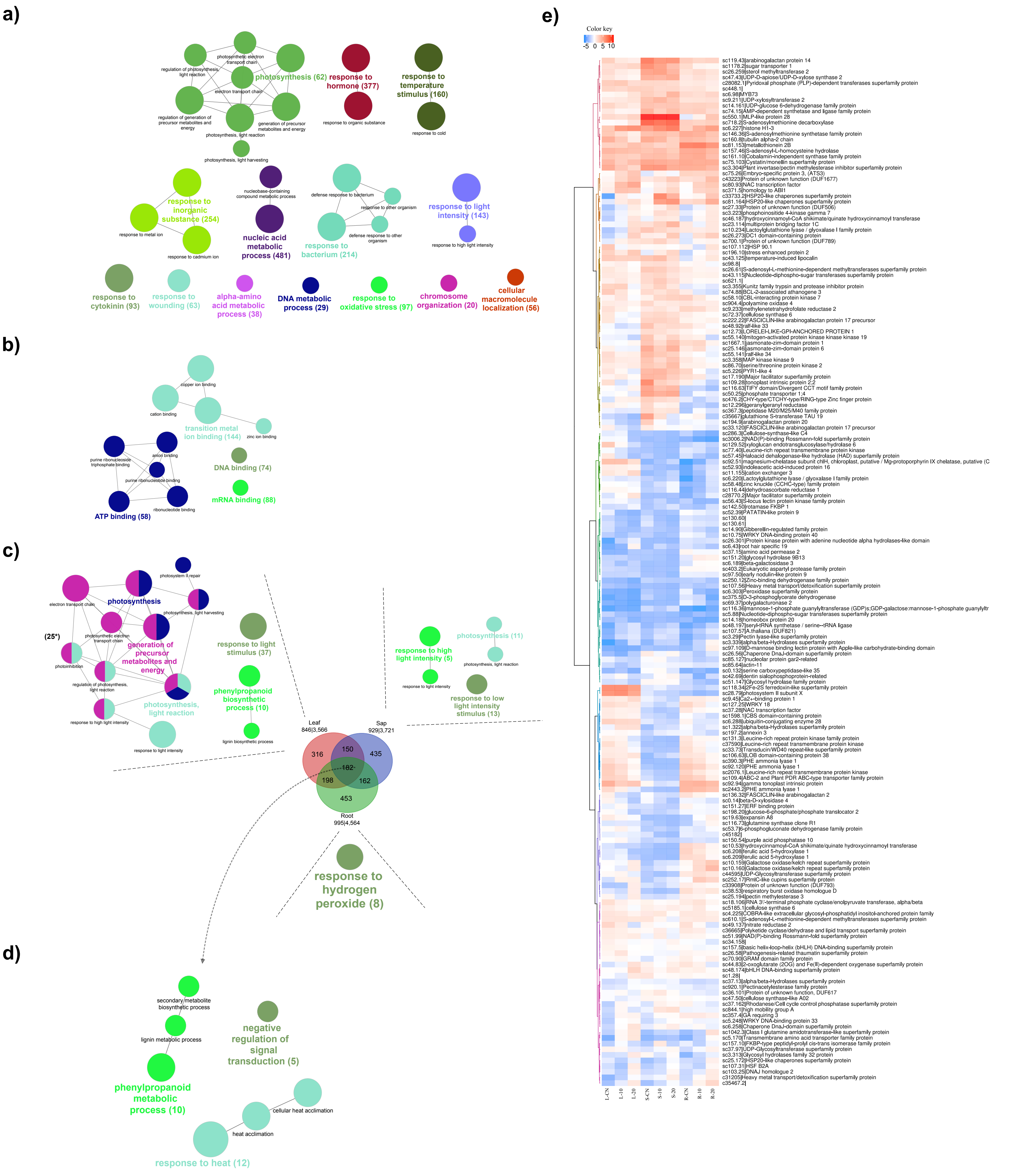

Supplement: Supplementary file 1 — Supplementary Figures [file 41598_2018_32904_MOESM1_ESM.pdf]
