# Supplementary material for: Transcriptomics and co-expression networks reveal tissue-specific responses and regulatory hubs under mild and severe drought in papaya (Carica papaya L.)
Source: Sci Rep. 2018 Sep 28;8:14539. doi: 10.1038/s41598-018-32904-2 (PMC6162326; doi:10.1038/s41598-018-32904-2)
Supplement: Supplementary file 2 — Supplementary Figures Legends [file 41598_2018_32904_MOESM2_ESM.docx]

**Title**

Transcriptomics and co-expression networks reveal tissue-specific responses and regulatory hubs under mild and severe drought in papaya (*Carica papaya* L.)

**Authors**

Gamboa-Tuz Samuel David^a,1^, Pereira-Santana Alejandro^a,1^, Zamora-Briseño Jesús Alejandro^a^, Castano Enrique^b^, Espadas-Gil Francisco^a^, Ayala-Sumuano Jorge Tonatiuh^c,d^, Keb-Llanes Miguel Ángel^a^, Sanchez-Teyer Felipe^a^, and Rodríguez-Zapata Luis Carlos^a,^*.

^a^ Biotechnology Unit, Yucatan Center for Scientific Research (CICY), 97205, Merida, Yucatan, Mexico.

^b^ Plant Biochemistry and Molecular Biology Unit, Yucatan Center for Scientific Research (CICY), 97205, Merida, Yucatan, Mexico.

^c^ IDIX S.A. de C.V. Av. Sonterra 3035 int. 26, Querétaro, México

^d^ Polytechnic University of Huatusco, 94100, Veracruz, México.

^1^ Contributed equally.

* Corresponding author. E-mail: lcrz@cicy.mx.

**Supplementary Figure S1. Distribution and annotation statistics of the assembled unigenes. a)** Length distribution and summary statistics of the assembled unigenes. **b)** Unigene BLAST Top-Hit distribution among the top 10 represented plant species from the PlantRef database of the NCBI. **c)** Unigene distribution across the most represented Level 3 Gene Ontology (GO) terms in each of the three GO categories: Biological Process (BP), Cellular Component (CC), and Molecular Function (MF). DESCRIPTION: The assembled transcriptome consisted of a total of 164,250 transcripts corresponding to 144,065 unigenes (**a**). About 35.7% (51,507) of the unigenes presented blast hits against the PlantRef protein database of the NCBI with the highest similarity to cacao (*Threobroma cacao*) and grape (*Vitis vinifera*) (**b**). Unigenes were annotated against the Gene Ontology (GO) Database (**c**). Most represented GO terms in BP were "Organic substance metabolic process", "Cellular metabolic process", and "Primary metabolic process." Additionally, "Nitrogen compound metabolic process" and "Response to stress" were represented. Most represented GO terms in CC were "Intracellular", "Intracellular part" and "Intracellular organelle". Most represented GO terms in MF were "Organic cyclic compound binding", "Heterocyclic compound binding", and "Ion binding." Additionally, "Hydrolase activity", and "Oxidoreductase activity" were represented (**c**).

**Supplementary Figure S2.** **Hierarchical Clustering and Principal Component Analysis of the 18 papaya cDNA libraries.** **a)** Heatmap and hierarchical clustering of the 18 papaya cDNA libraries based on pairwise Pearson's correlation coefficient of their expression values, estimated by the reference-based transcriptomic analysis. **b)** Heatmap and hierarchical clustering of the 18 papaya cDNA libraries based on pairwise Pearson's correlation coefficient of their expression values, estimated by the *de novo* transcriptomic analysis. **c)** Principal component analysis (PCA) of the 18 cDNA libraries based on expression values, estimated by the reference-based transcriptomic analysis. **d)** PCA of the 18 cDNA libraries based on expression values, estimated by the *de novo* transcriptomic analysis. In a)-d) expression values were analyzed as counts per million (CPM). In a) and b) the dendrograms were calculated by the "euclidean" distance and the "complete" clustering methods. In c) and d) only the first two components are plotted. Samples and replicates are described in **Table 1** (main text).

**Supplementary Figure S3. Differentially expressed genes among leaf, sap, and roots under control (CN) condition** **identified in the reference-based transcriptomic analysis**. **a)** Volcano plot comparing S-CN_vs_R-CN. **b)** Volcano plot comparing L-CN_vs_R-CN. **c)** Volcano plot comparing S-CN_vs_L-CN. **d)** Venn diagram depicting the intersection of up-regulated genes in leaves (L-CN), sap (S-CN), and roots (R-CN) under control condition. In a)-c) Up-regulated genes (absolute FC ≥ 2 and FDR value ≤ 0.001) are in red, blue, and green for leaves, sap, and roots, respectively; genes that were not differentially expressed (unchanged) are in gray. L = Leaf, S = Sap, and R = Root. Sample names are described in **Table 1** (main text), and individual FC and FDR values are presented in **Supplementary Table S4**.

**Supplementary Figure S4. Drought-responsive differentially expressed genes identified in the reference-based transcriptomic analysis. a)** Volcano plot comparing L-CN_vs_L-10. **b)** Volcano plot comparing L-CN_vs_L-20. **c)** Volcano plot comparing S-CN_vs_S-10. **d)** Volcano plot comparing S-CN_vs_S-20. **e)** Volcano plot comparing R-CN_vs_R-10. **f)** Volcano plot comparing R-CN_vs_R-20. Up-regulated genes (FC ≥ 2 and FDR ≤ 0.001) are in red, and down-regulated genes (FC ≤ -2 and FDR ≤ 0.001) are in blue; genes that were not differentially expressed are in gray. CN = Control condition, L = Leaf, S = Sap, and R = Root. Samples names are described in **Table 1** (main text), and FC and FDR values are presented in **Supplementary Table S4**.

**Supplementary Figure S5. Clustering of the drought-responsive differentially expressed genes and unigenes in response to drought.** **a)** Heatmap and hierarchical clustering of the 18 cDNA libraries and the 8,549 DDEGs identified in the reference-based transcriptomic analysis based on their TPM values (mean-centered log2(TPM+1)). **b)** Expression patterns of the clusters of DDEGs identified by hierarchical clustering in a). **c)** Heatmap and hierarchical clustering of the 18 cDNA libraries and the 6,089 DDEUs identified in the *de novo* transcriptomic analysis based on their TPM values (mean-centered log2(TPM+1)). **d)** Expression patterns of the clusters of DDEUs identified by hierarchical clustering in c). In a) and c) libraries (columns) and genes/unigenes (rows) were clustered by means of the "euclidean" distance and "complete" clustering methods. In b) and d) mean values are indicated by black points connected with a black line. Sample and replicate names are described in **Table 1** (main text).

**Supplementary Figure S6. Differentially expressed unigenes among leaf, sap, and roots under control (CN) condition** **identified in the *de novo* transcriptomic analysis**. **a)** Volcano plot comparing S-CN_vs_R-CN. **b)** Volcano plot comparing L-CN_vs_R-CN. **c)** Volcano plot comparing S-CN_vs_L-CN. **d)** Venn diagram depicting the intersection of up-regulated unigenes in leaves (L-CN), sap (S-CN), and roots (R-CN) under the control condition. In a)-c) Up-regulated unigenes (absolute FC ≥ 2 and FDR value ≤ 0.001) are in red, blue, and green for leaves, sap, and roots, respectively; unigenes that were not differentially expressed (unchanged) are in gray. L = Leaf, S = Sap, and R = Root. Sample names are described in **Table 1** (main text), and FC and FDR values are presented in **Supplementary Table S5**.

**Supplementary Figure S7. Drought-responsive differentially expressed unigenes identified in the *de novo* transcriptomic analysis. a)** Volcano plot comparing L-CN_vs_L-10. **b)** Volcano plot comparing L-CN_vs_L-20. **c)** Volcano plot comparing S-CN_vs_S-10. **d)** Volcano plot comparing S-CN_vs_S-20. **e)** Volcano plot comparing R-CN_vs_R-10. **f)** Volcano plot comparing R-CN_vs_R-20. Up-regulated unigenes (FC ≥ 2 and FDR ≤ 0.001) are in red, and down-regulated unigenes (FC ≤ -2 and FDR ≤ 0.001) are in blue; unigenes that were not differenitally expressed are in gray. CN = Control condition, L = Leaf, S = Sap, and R = Root. Sample names are described in **Table 1** (main text), and FC and FDR values are presented in **Supplementary Table S5**.

**Supplementary Figure S8. Intersection of differentially expressed gene models identified in the reference and *de novo* transcriptomic analyses.** Unigenes were blasted against the gene models of the reference genome of the transgenic 'Sunup' papaya for comparison. **a)** Intersection of DEGs in control conditions (L-CN, S-CN, and R-CN). **b)** Intersection of DDEGs in leaves at 10 (L-10) and 20 (L-20) DASI. **c)** Intersection of DDEGs in sap at 10 (S-10) and 20 (S-20) DASI. **d)** Intersection of DDEGs in roots at 10 (R-10) and 20 (R-20) DASI. In a)-d) the percentage of gene models present in the *de novo* transcriptomic analysis (identified by blast searches) indicated in parenthesis in the intersection area. Sample names are described in **Table 1** (main text).

**Supplementary Figure S9. Specific core sets of DDEGs identified in the reference-based transcriptomic analysis. a)** Core sets of DDEGS that were simultaneously up-regulated or down-regulated at both 10 DASI and 20 DASI, per tissue type. **b)** Core sets of DDEGs that were simultaneously up-regulated or down-regulated in all three tissues (leaves, sap, roots), at 10 DASI or 20 DASI. The DDEGs from these core sets, from both a) and b), can be inspected in **Supplementary Table S2**.

**Supplementary Figure S10. ClueGO clustering analysis over the enriched GO terms found by Blast2GO in the tissue-specific up-regulated gene sets under control (CN) condition. a)** Enriched GO groups in L-CN, **b)** Enriched GO groups in S-CN, **c)** Enriched GO groups in R-CN. Biological Process (BP) GO category was used to the clustering analysis. Colored circles are used only to facilitate cluster identification. Sample names and treatments are described in **Table 1** (main text).

**Supplementary Figure S11. ClueGO clustering analysis over the enriched GO terms found by Blast2GO in the DDEGs sets**. **a)** Enriched GO groups in up-regulated DDEGs set in L-10. **b)** Enriched GO groups in up-regulated DDEGs set in S-10. **c)** Enriched GO groups in up-regulated DDEGs set in R-10. **d)** Enriched GO groups in up-regulated DDEGs set in L-20. **e)** Enriched GO groups in up-regulated DDEGs set in S-20. **f)** Enriched GO groups in up-regulated DDEGs set in R-20. **g)** Enriched GO groups in down-regulated DDEGs set in L-10. **h)** Enriched GO groups in down-regulated DDEGs set in S-10. **i)** Enriched GO groups in down-regulated DDEGs set in R-10. **j)** Enriched GO groups in down-regulated DDEGs set in L-20. **k)** Enriched GO groups in down-regulated DDEGs set in S-20. **l)** Enriched GO groups in down-regulated DDEGs set in R-20. Biological Process (BP) GO category was used to the clustering analysis. Colored circles are used only to facilitate cluster identification. Sample names and treatments are described in **Table 1** (main text).

**Supplementary Figure S12. Gene co-expression networks of the up-regulated DDEGs of papaya leaves during drought treatments.** Network statistics and gene names are shown. The 37 most connected TFs reported in abiotic stress were used as regulators of the network. Numbers of genes (nodes) up-regulated at 10 DASI, 20 DASI, and BOTH stress treatments are indicated. Description of all nodes (genes) are presented in **Supplementary Tables S7**.

**Supplementary Figure S13. Gene co-expression networks of the up-regulated DDEGs of papaya roots during drought treatments. Network statistics and gene names are shown.** The 56 most connected TFs reported in abiotic stress were used as regulators of the network. Numbers of genes (nodes) up-regulated at 10 DASI, 20 DASI, and BOTH stress treatments are indicated. Description of all nodes (genes) are presented in **Supplementary Tables S8**.

**Supplementary Figure S14. Heatmaps of Up-regulated and Down-regulated DDEGs found within any enriched GO term related to water deprivation or abscisic acid (ABA). a)** 142 up-regulated DDEGs and **b)** 192 Down-regulated DDEGs. These DDEGs can be inspected in **Supplementary Table S2**. DESCRIPTION: from **Supplementary Table S6**, we took the IDs of the genes that were found within any enriched GO term containing the words “water deprivation” or “abscisic acid” in any of the stressed libraries (L-10, L-20. S-10, S-20, R-10, and R-20). In a) and b) "sc" = “evm.TU.supercontig_” and “c” = “evm.TU.contig_”. We only included DDEGs that were up-regulated in any stressed library, but not down-regulated in any other stressed library, and vice versa. It is worth noting that these genes may be associated to other GO terms, and that many other genes associated with the depicted GO terms may be present in our transcriptome, but they were not within any enriched GO term in the stressed libraries (so they were not depicted). Dendrograms were generated with the Euclidean distance and the complete clustering methods. Color key indicates mean-centered log2(TPM+1) of the mean values of replicates per library. Enriched GO terms: ABAMP = abscisic acid metabolic process (GO:0009687), NRABASP = negative regulation of abscisic acid-activated signaling pathway (GO:0009788), RABA = response to abscisic acid (GO:0009737), RWD = response to water deprivation (GO:0009414), ABAASP = abscisic acid-activated signaling pathway (GO:0009738), CRWD = cellular response to water deprivation (GO:0042488), OS = response to osmotic stress (GO:0006970).

**Supplementary Figure S15. ClueGO clustering analysis of total mobile mRNA and Heatmap of mobile DDEGs found in papaya plant.** A total of 4,408 papaya genes (mRNAs) were identified as “mobile” based on homology comparisons (blast searches) against all of the 5,234 Arabidopsis mobile mRNAs from the Plant Mobile Macromolecules database (PlaMoM; <http://www.systembioinfo.org/plamom/>). **a)** ClueGo clulstering analysis by Biological Process (BP) of the total 4,408 identified mobile genes (mRNAs) in papaya. **b)** ClueGO clustering analysis by Molecular Function (MF) of the total 4,408 identified mobile genes (mRNAs) in papaya. **c)** Venn diagram and ClueGO clustering (by BP) of the specific sets of mobile DDEGs detected in each studied sample: 866 for leaves (L-10 + L-20), 929 for sap (S-10 + S-20), and 995 for roots (R-10 + R-20). **d)** ClueGO clustering by BP of the shared core set of 182 mobile DDEGs (this core set can be inspected in **Supplementary Table S2**, “CORE_mobile” column) in the three tissues as depicted in c). **e)** Heatmap of the shared core set of 182 mobile DDEGs (as depicted in c) and d)) in papaya. Color key in e) indicates mean-centered log2(TPM+1) of the mean values of replicates per library; the dendrogram on the left was generated with the “maximum” distance and the “ward” clustering methods. Colored circles in a)-d) are used only to facilitate cluster identification. Venn diagram was depicted using the Venn diagrams software (<http://bioinformatics.psb.ugent.be/webtools/Venn/>) from the Bioinformatics and Evolutionary Genomics Lab (UGENT, Belgium). DDEG = drought-responsive differentially expressed genes.
